# Melem‐Perylene Diimide Polymer Network as Efficient Positive Electrode for Rechargeable Lithium and Magnesium Batteries

**DOI:** 10.1002/cssc.202500967

**Published:** 2025-09-08

**Authors:** Ruth Gomes, Jan Kraus, Igor Krivtsov, Vivek Wakchaure, Sibylle Riedel, Zhirong Zhao‐Karger, Johannes Liessem, Christof Neumann, Martin Oschatz, Andrey Turchanin, Maximilian Fichtner, Radim Beranek, Max von Delius

**Affiliations:** ^1^ Institute of Organic Chemistry Ulm University Albert‐Einstein‐Allee 11 89081 Ulm Germany; ^2^ Department of Chemical and Environmental Engineering University of Oviedo Avenida Julián Clavería 8 33006 Oviedo Spain; ^3^ Institute of Electrochemistry Ulm University Albert‐Einstein‐Allee 47 89081 Ulm Germany; ^4^ Helmholtz Institute Ulm (HIU) Helmholtzstraße 11 89081 Ulm Germany; ^5^ Institute of Nanotechnology Karlsruhe Institute of Technology Hermann‐von‐Helmholtz‐Platz 1 76344 Eggenstein‐Leopoldshafen Germany; ^6^ Institute of Physical Chemistry Friedrich Schiller University Jena Lessingstraße 10 07743 Jena Germany; ^7^ Institute for Technical Chemistry and Environmental Chemistry Friedrich Schiller University Jena Philosophenweg 7a 07743 Jena Germany; ^8^ Helmholtz Institute for Polymers in Energy Applications Jena (HIPOLE Jena) Lessingstraße 12–14 07743 Jena Germany; ^9^ Center for Energy and Environmental Chemistry Jena (CEEC Jena) Philosophenweg 7a 07743 Jena Germany

**Keywords:** cathode materials, Li‐ion batteries, Mg batteries, organic electrodes, polymer networks

## Abstract

Organic battery electrode materials represent a sustainable alternative compared to most inorganic electrodes, yet challenges persist regarding their energy density and cycling stability. In this work, a new organic electrode material is described, which is obtained via ionothermal polymerization of low‐cost starting materials, melem (2,5,8‐triamino‐tri‐*s*‐triazine) and perylenetetracarboxylic dianhydride (PTCDA). The resulting networked polymer **Melem‐PDI** exhibits favorable thermal and electrochemical properties, prompting investigation into its performance as a positive electrode material in rechargeable lithium and magnesium batteries. A hybrid material with carbon nanotubes (**Melem‐PDI‐CNT**) is found to exhibit‐excellent cycling stability in Li‐ion batteries at a current rate as high as 500 mA g^−1^ for 5000 cycles. While the Li‐ion storage is based on a pseudocapacitive mechanism, a diffusion‐controlled mechanism is observed in magnesium batteries. This work underscores that classic dyes (here: PDI) can be repurposed for energy storage, once they are integrated into suitable polymer topologies and brought into nanoscale contact with conductive materials.

## Graphical abstract

An organic electrode combining perylene diimide and heptazine was synthesized in one pot and tested as a positive electrode for lithium and magnesium batteries. The polymer network offers high theoretical capacity. Lithium cells exhibit excellent cycling stability, while magnesium, as a postlithium system, shows promising performance.

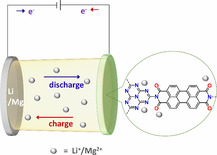

## 1 Introduction

Lithium‐ion batteries (LIBs) have revolutionized energy storage technology since their first commercialization in 1991, powering devices from smartphones to electric vehicles thanks to their long cycle life and high energy density.^[^ ^1^, ^2^ ^]^ Despite the remarkable performance of LIBs, there is still room for improvement, especially in terms of power density and cycling stability.^[^ ^3^, ^4^ ^]^ Additionally, the raw materials used in commercial inorganic electrodes of LIBs raise concerns regarding their cost and limited availability, as well as the environmental impact of waste disposal.^[^ ^5^, ^6^ ^]^ Organic electrode materials offer a sustainable, low‐cost, and environmentally friendly alternative.^[^ ^7^, ^8^, ^9^ ^]^ Furthermore, organic materials are also attractive thanks to the possibility of fine‐tuning at the molecular level performance aspects of specific battery systems.^[^ ^10^, ^11^, ^12^, ^13^, ^14^, ^15^, ^16^ ^]^ One disadvantage of using organic small molecules as electrode material for batteries is the dissolution of electrode active material into the electrolyte. This can be overcome by transforming the redox‐active molecule into an insoluble linear macromolecule or polymeric network.^[^ ^17^, ^18^, ^19^ ^]^ Another concern regarding organic electrode materials is their poor intrinsic electronic conductivity, which requires the addition of large amounts of conductive additives such as carbon black, graphene, or carbon nanotubes (CNT). Beyond this mechanical mixing of organic materials with conductive carbon, elegant strategies to increase the intrinsic conductivity include the preparation of hybrid materials with nanoscale integration of conductive components or the use of aromatic building blocks.^[^ ^20^, ^21^, ^22^, ^23^, ^24^, ^25^, ^26^, ^27^ ^]^

Besides the exploration of organic electrode materials, researchers in the field are also aiming to address inherent limitations of lithium‐ion technology by studying different charge carriers. Among possible “postlithium” technologies, rechargeable magnesium batteries (RMB) stand out due to their higher theoretical capacity, increased safety, and the abundance of magnesium as a raw material.^[^ ^28^ ^]^ The divalent Mg^2+^ ions possess an ionic radius comparable to that of the Li^+^ ions, yet carry twice the charge density. In the fabrication of Mg batteries, organic positive electrode materials have been proven to be advantageous over conventional inorganic intercalation cathodes generally used for LIBs.^[^ ^29^, ^30^ ^]^ This is mainly due to the sluggish kinetics of the high‐charge‐density divalent Mg^2+^ ion insertion/deinsertion into the host materials via the classic “rocking‐chair” mechanism.^[^ ^31^, ^32^ ^]^ Conversion cathodes such as sulfur and redox‐active organic materials can provide reversible charge storage via the redox reaction of the functional groups, which can also be tuned to offer multiple electron reactions.^[^ ^33^, ^34^, ^35^, ^36^ ^]^ A combination of a redox‐active organic cathode with a suitable non‐nucleophilic Mg‐electrolyte can provide a highly stable and efficient organic Mg battery, leading to the advancement toward a cost‐effective and environmentally benign battery technology.^[^ ^37^, ^38^, ^39^ ^]^

In this work, we report the one‐step synthesis of a novel organic polymer network, named **Melem‐PDI**, and its application as a positive electrode material for rechargeable Li and Mg batteries. The hybrid material we obtained by performing the synthesis in the presence of multiwalled CNT (**Melem‐PDI‐CNT**) exhibits excellent cycling stability against lithium for 5000 cycles at a current rate of 500 mA g^−1^, while the corresponding magnesium battery shows promising characteristics, but still lacks long‐term cyclability. Perylene diimide (PDI) was chosen as the key organic building block because of its low cost and potential to increase the inherent conductivity of the material as an aromatic, organic semiconductor.^[^ ^40^, ^41^ ^]^ Related PDI‐based materials have recently been employed as electrode materials in Li and Mg batteries, yet this work stands out due to the ease of synthesis and excellent cycling performance without significant loss in the specific capacity when employed as a positive electrode.^[^ ^42^, ^43^, ^44^ ^]^

## 2 Results and Discussion

### 2.1 Synthesis and Characterization

**Figure 1:**
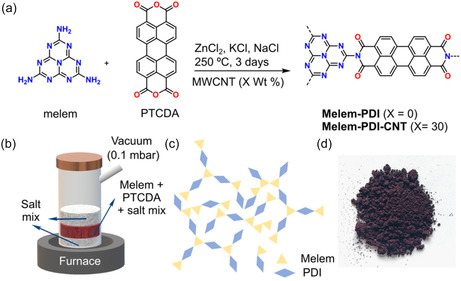
a) Synthesis of **Melem‐PDI** and **Melem‐PDI‐CNT** material, b) schematic representation of the synthesis protocol, c) schematic illustration of the structure of the **Melem‐PDI** material, and d) photograph of the **Melem‐PDI** powder.

The **Melem‐PDI** material was synthesized via ionothermal (poly)condensation reaction between the bulk chemical perylenetetracarboxylic dianhydride (PTCDA, CAS: 128‐69‐8) and melem (2,5,8‐triamino‐tri‐*s*‐triazine) in a molten mixture of zinc (II) chloride, potassium chloride, and sodium chloride at 250 °C over 3 days (**Figure** **1**a, synthesis details in Supporting Information).^[^ ^45^, ^46^, ^47^ ^]^ A schematic representation of the reaction setup and the topology of the **Melem‐PDI** polymeric network is shown in Figure 1b,c.

**Melem‐PDI** was obtained as a dark purple powder in 85% yield (Figure 1d). To endow the envisaged electrode material with enhanced conductivity, we also carried out the same synthesis procedure in the presence of multiwalled CNT (MWCNT, 30 wt%), furnishing the hybrid material **Melem–PDI–CNT** in 94% yield. Both materials and precursors were characterized with a variety of methods to establish their structure, morphology, and chemical composition.

**Figure 2:**
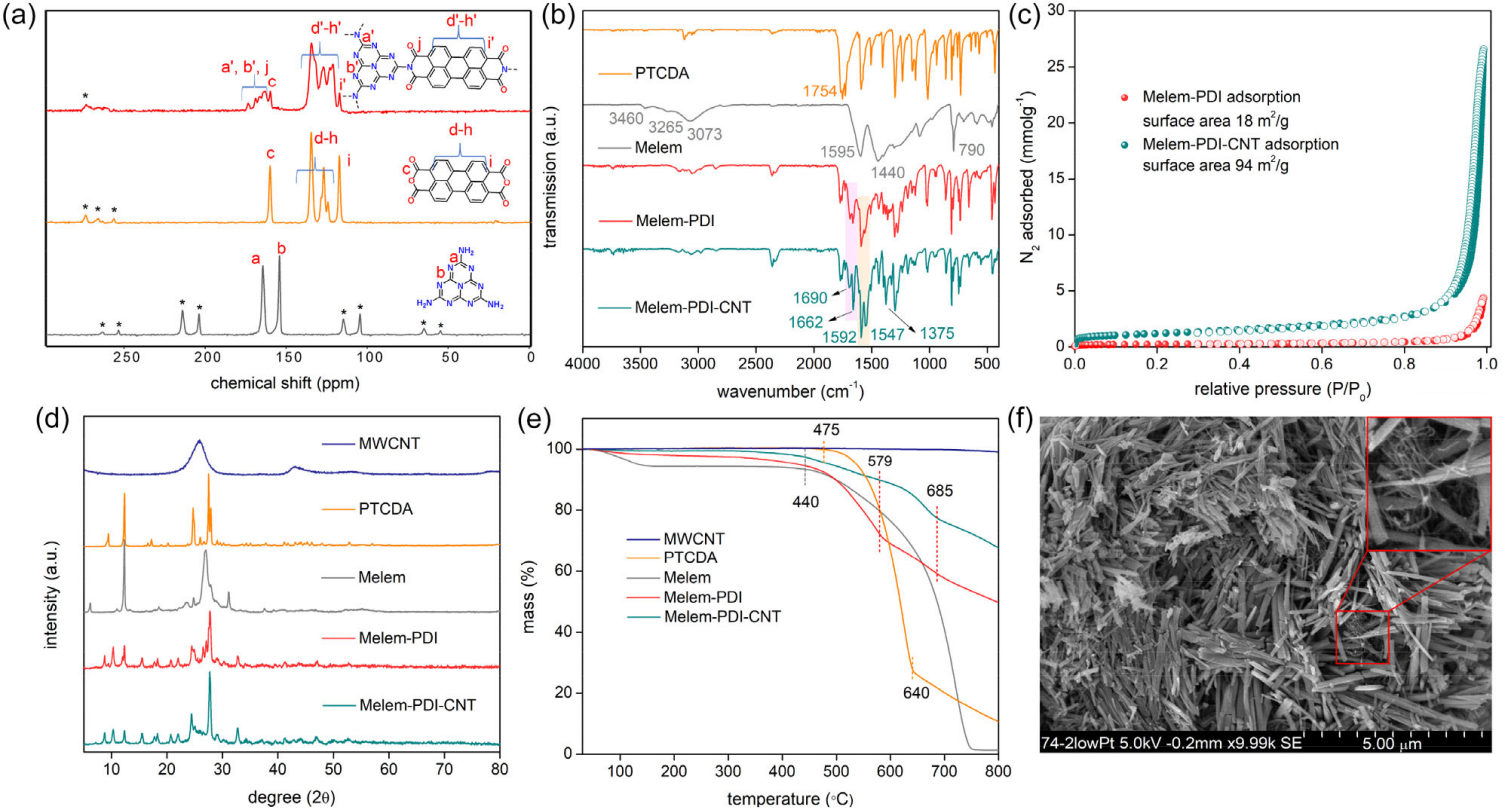
a) Solid‐state ^13^C CPMAS NMR spectra of the starting materials and **Melem‐PDI** (14 kHz, 298 K) (*'s indicate spinning sidebands); b–e) FT‐IR spectra, N_2_ adsorption/desorption isotherm, PXRD pattern, and TGA profile under N_2_ for both materials and the precursors; f) scanning electron microscopic image of the **Melem‐PDI‐CNT** hybrid material (presence of CNTs is shown in the zoomed area). (a.u.–arbitrary units).

**Figure** **2**a shows the solid‐state ^13^C cross‐polarization and magic angle spinning (CP‐MAS) nuclear magnetic resonance (NMR) spectrum of **Melem‐PDI**. The spectrum includes broad signals in two distinct regions: 117 − 135 ppm and 160 − 173 ppm. Based on the CP‐MAS NMR spectra of the two starting materials (Figures S1 and S3, Supporting Information), we assign the signals at higher field to the aromatic carbon atoms of the perylene unit, whereas the signals at lower field are assigned to the imide carbonyl carbons and the carbon atoms from the melem unit. ^15^N MAS NMR spectra of melem and the **Melem‐PDI** material (Figures S5, Supporting Information) provide clear evidence for successful polymerization, as the signal corresponding to free amine (—NH_2_) nitrogen atoms at 107.87 ppm disappears.

Fourier transform infrared spectra (FT‐IR, Figure 2b) corroborate the near‐complete disappearance of peaks attributed to primary amine (3460, 3265, and 3073 cm^−1^) in the **Melem‐PDI** and **Melem‐PDI‐CNT**, indicating a relatively low level of defects occurring from incomplete polymerization.^[^ ^48^ ^]^ Moreover, the intensity of the anhydride carbonyl stretching band of the **PTCDA** starting material (1754 cm^−1^) decreases, and the emergence of a new band for the imide carbonyl at 1690 and 1662 cm^−1^, in the **Melem‐PDI** and the **Melem‐PDI‐CNT** materials, supports the formation of imide linkages. The ionothermal synthesis previously reported by Lotsch^[^ ^45^ ^]^ appeared particularly promising in a survey of polymerization conditions (Scheme S1, Supporting Information). A residual absorption band near 1755 cm^−1^ is observed, likely due to the presence of unreacted anhydride groups at the polymer chain ends. This observation can be attributed to the low inherent reactivity of melem and to the absence of an end‐capping procedure. The peaks at 1375 cm^−1^ in the **Melem‐PDI** and the **Melem‐PDI‐CNT** materials can be assigned to the stretching vibration of the imide C—N bond, while that at 1592 and 1547 cm^−1^ is attributed to the C=N and C=C stretching, respectively.

Elemental (CHN) analysis (Table S1, Supporting Information) confirms the incorporation of **PTCDA**, as evidenced by a significant increase in the C/N ratio. X‐ray photoelectron spectroscopy (XPS) was employed to investigate the surface composition of **Melem‐PDI** and **Melem‐PDI‐CNT**. The XP survey spectra of the precursor materials, **Melem** and **PTCDA**, are consistent with previously reported literature values (Figure S7–S8, Supporting Information)^[^ ^49^, ^50^, ^51^ ^]^ In the high resolution (HR) C 1s XP spectra of both **Melem‐PDI** and **Melem‐PDI‐CNT**, a new peak at 288 eV was observed, corresponding to C=O groups from imide units. Quantitative XPS analysis (Figure S9, S10, Table S2, Supporting Information) shows a substantial increase in the C/N ratio from 0.84 (**Melem**) to 4.75 (**Melem‐PDI**) and 7.67 (**Melem‐PDI‐CNT**), similar to the CHN elemental analysis results. The overall decrease in nitrogen and oxygen content on the surface, as well as the presence of characteristic binding features, indicates the formation of covalent bonds between the **PTCDA** units and **Melem** (Table S3, S4, Supporting Information). Additionally, a new, weak signal in the HR‐N 1s spectra at 400 eV was attributed to imide nitrogen atoms (imide–N), associated with the O=C—N—C=O bonding environment (Figure S9, S10, Supporting Information).^[^ ^52^ ^]^ This provides strong evidence for successful imide formation during polymerization.

Figure 2c depicts the N_2_ adsorption–desorption isotherm of both materials, where filled circles indicate adsorption and empty circles indicate desorption points. The isotherm for the **Melem‐PDI** material shows a surface area of 18 m^2^ g^−1^ and a pore volume of 0.15 cm^3 ^g^−1^, suggesting a low intrinsic porosity of the material. The **Melem‐PDI‐CNT** material shows a similar isotherm along with a hysteresis in the high‐pressure region, possibly due to N_2_ condensation occurring in the interparticle voids. Further, the incorporation of the CNT has resulted in an increase in the surface area (94 m^2^ g^−1^) and pore volume (0.92 cm^3^ g^−1^), which can be beneficial for the battery performance of the **Melem‐PDI‐CNT** electrodes.

The crystallinity of the synthesized **Melem‐PDI** and **Melem‐PDI‐CNT** materials, as well as the precursor molecules, was investigated utilizing powder X‐ray diffraction (PXRD), and the results are shown in Figure 2d. The PXRD analysis confirms the structure of PTCDA.^[^ ^53^ ^]^ Notably, the PXRD pattern of melem differs from the one reported by Schnick et al.^[^ ^46^ ^]^ This can be readily explained since our synthesis involved washing of the melem sample in water, during which it underwent a reassembly into H‐bonded melem network, thus yielding a similar PXRD pattern as reported by Shalom et al.^[^ ^54^ ^]^ The reflexes corresponding to the individual phases of Melem and PTCDA in the diffractogram of the **Melem‐PDI** sample show significant broadening and significantly reduced intensities, while a series of new diffraction reflexes appears (Figure 1d), confirming the synthesis of a novel **Melem‐PDI** material. The nearly identical PXRD patterns of **Melem‐PDI** and **Melem‐PDI‐CNT** materials demonstrate that the crystalline structure of **Melem‐PDI** is retained upon the synthesis in the presence of 30 wt% MWCNTs. Moreover, the broad MWCNT reflex is still present in **Melem‐PDI‐CNT** below the rather sharp reflexes belonging to **Melem‐PDI**. The absence of the characteristic first intense diffraction peak of the ordered 2D lattice in layered covalent organic frameworks (COFs) indicates that the material obtained is not a COF (Figure S11, Supporting Information). Instead, the structure is more consistent with low‐dimensional assemblies, which is sufficient to produce some crystalline features in PXRD and small‐angle X‐ray scattering. Figure 2e shows the thermogravimetric analysis (TGA) weight‐loss profiles of both materials and the starting components from room temperature to 800 °C at 10 °C min^−1^ under nitrogen atmosphere (see Figure S12, Supporting Information for TGA under oxygen). The pristine MWCNT sample shows no significant weight loss in the measured temperature window under nitrogen, whereas the melem starting material exhibits thermal stability up to 440 °C and degrades fully up to 750 °C. The PTCDA starting material demonstrates a two‐step weight loss at 475 and 640 °C. Both the **Melem‐PDI** and **Melem‐PDI‐CNT** samples show three‐step weight loss corresponding to stepwise decomposition of the Melem and PTCDA building blocks. The most pronounced difference between the **Melem‐PDI** sample and its precursors is that it does not undergo complete degradation upon heating to 800 °C (Figure 2e, red line), which is likely due to its partial carbonization under a reductive atmosphere. The higher residual weight of **Melem‐PDI‐CNT** (68%) compared to **Melem‐PDI** (50%) can be attributed to the **CNT** content in **Melem‐PDI‐CNT**. Since 30% **CNT** was added during synthesis and 68% residual mass was determined, we deduce that the **CNT** remains unchanged during hybrid material synthesis.

A bathochromic shift of ≈50 nm is observed in the UV–visible absorption spectrum of Melem‐PDI compared to its precursor, PTCDA (Figure S13, Supporting Information), indicating expansion of the π‐conjugation system. The scanning electron microscope (SEM) image of the **Melem‐PDI‐CNT** material (Figure 2f and Figure S14, Supporting Information) shows uniform needlelike morphology with small clusters that could be indicative of bundles of MWCNT (Figure 2f, inset) throughout the framework. The more uniform formation of the needlelike particles as compared to the flakes of the **Melem‐PDI** material (as seen in Figure S14a, S14b, Supporting Information) is likely due to the in situ incorporation of MWCNT, which acts like a seed or template for the synthesis of the polymer particles.

The average dimension of the Melem‐PDI‐CNT particles was 140 nm × 2.4 μm, whereas that of the Melem‐PDI particles was 390 nm × 2.3 μm. The transmission electron microscopic image (Figure S15, Supporting Information) of the Melem‐PDI material also shows rectangular flake‐like particles with an average dimension of 125 nm × 750 nm.

### 2.2 Electrochemical Characterization and Properties for Li‐Ion Storage

**Figure 3:**
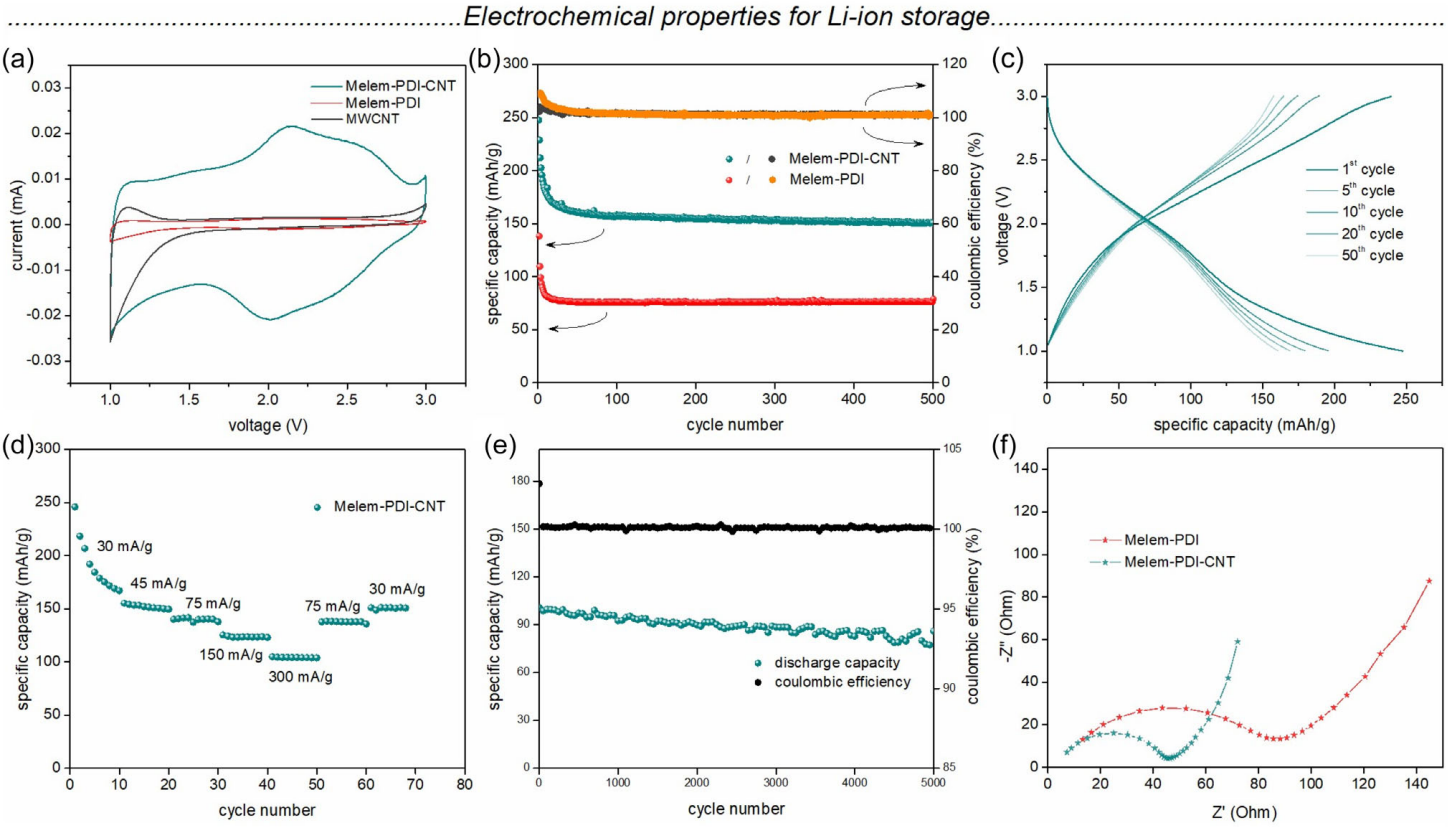
a) CV of **Melem‐PDI‐CNT**, **Melem‐PDI, and MWCNT** at a scan rate of 0.1 mV s^−1^, b) battery cycling performance of **Melem‐PDI‐CNT** and **Melem‐PDI** at a current rate of 50 mA g^−1^, c) galvanostatic charge–discharge profile for selected cycles, and d) rate capability performance of **Melem‐PDI‐CNT**, e) cycling stability of **Melem‐PDI‐CNT** at a current rate of 500 mA g^−1^, and f) electronic impedance spectra of both materials against Li anode in 1 M LiTFSI electrolyte dissolved in 1:1 DOL:DME.

The electrochemical characterization and the battery performance of the **Melem‐PDI** material and the **Melem‐PDI‐CNT** hybrid material were first investigated in Li‐based half cells. The electrodes were cast on a carbon‐coated aluminum foil with a slurry containing 7:2:1 weight ratio of active material, conductive carbon black, and polyvinylidene fluoride binder in NMP solvent (see Supporting Information for details). 1 M lithium bis(trifluoromethanesulfonyl)imide (LiTFSI) + 0.25 M LiNO_3_ in a 1:1 (v:v) mixture of dioxolane and 1,2‐dimethoxyethane was used as the electrolyte. **Figure** **3**a shows the cyclic voltammogram (CV) of **MWCNT**, **Melem‐PDI** (red), and **Melem‐PDI‐CNT** (teal) at a scan rate of 0.1 mVs^−1^ in the potential window of 1–3 V versus Li/Li^+^. Although both polymers demonstrate a reversible broad redox‐couple with the reduction peak centered at ≈2 V and the corresponding oxidation peak near 2.25 V,^[^ ^55^, ^56^ ^]^ the current for the **Melem‐PDI‐CNT** is significantly increased across the voltage range (by factor ca.16) compared to the **Melem‐PDI** electrode. The CV of **MWCNT,** on the other hand, does not show any significant redox peak in the relevant voltage window (Figure 3a, black line). Rather, a flat CV profile corresponding to insignificant capacitive storage can be observed. A zoom into the CV profiles of the **Melem‐PDI** and **MWCNT** electrodes is shown in Figure S18, Supporting Information, for better comparison of their redox behavior. An investigation of the CV profiles of both the positive electrodes at various scan rates and the corresponding log–log plot of the peak current (*i*) versus the scan rate (*υ*) is provided in Figure S19, Supporting Information. The *b* values (following the equation *i *= *aυ* ^*b*^) obtained from both the cathodic and anodic peaks for both of the materials are close to unity, indicating a pseudocapacitive/surface‐controlled storage mechanism of the Li‐ions into the material framework. This result is in agreement with the observed capacitive behavior in the CV profiles (Figure 3a) and the capability to charge with high rates (Figure 3d).

Further, galvanostatic charge–discharge cycling was performed with both of the cathodes at a current rate of 50 mA g^−1^ in the potential window of 1–3 V. After an initial capacity loss in the first few cycles, the cells show very stable specific discharge capacity values for 500 cycles (Figure 3b). The specific discharge capacity values after 500 cycles are 151 and 77 mAh g^−1^ for **Melem‐PDI‐CNT** and **Melem‐PDI** cathodes, respectively. Figure 3c shows the galvanostatic charge–discharge profile for selected cycles of the **Melem‐PDI‐CNT** electrode. The slopy nature of the plateaus matches the broad cathodic and anodic peaks in the CV. The irreversible capacity loss in the initial cycles can be attributed to some irreversible consumption of lithium. To obtain insights into the mechanism of Li‐ion insertion into the polymeric network, we performed ex situ FT‐IR spectroscopy of the pristine electrode as well as after one complete discharge and charge (Figure S20, Supporting Information). The reduction of the peak intensity at 1592 cm^−1^ (C=N) and at 1660 cm^−1^ (C=O) after discharge can be associated with the lithiation of the imide carbonyl as well as the heptazine unit. Interestingly, one additional peak appears at 864 cm^−1^ after discharge, which can be assigned to the C—N—Li bond, suggesting the participation of the heptazine unit in the lithiation process during battery cycling. The participation of the melem unit in the redox process could also explain the capacitive storage mechanism, which differs from the Faradaic redox reaction of typical polyimide‐based materials in the Li system.^[^ ^57^ ^]^

A plausible reaction mechanism of the lithiation/delithiation process occurring during the discharge and charge process, showing 2e^−^ transfer per PDI unit and 6e^−^ transfer per melem unit, is illustrated in Figure S16 and Figure S17, Supporting Information.^[^ ^58^ ^]^

The theoretical capacity of the **Melem‐PDI** material was calculated to be 320 mAh g^−1^ (see Supporting Information for details), assuming a 3e^−^ redox reaction occurring per repeat unit of the polymeric network, whereas it is 213 mAh g^−1^ assuming a 2e^−^ redox process. Further, the rate capability performance of the **Melem‐PDI‐CNT** hybrid material for the lithium‐ion storage was investigated (Figure 3d). The cathode delivered average specific discharge capacities of 178, 152, 140, 123, and 105 mAh g^−1^ for increasing current rate starting from 30 to 300 mA g^−1^, respectively. The specific discharge capacity further increased to 138 and 151 mAh g^−1^ when the current density was again increased to 75 and 30 mA g^−1^, respectively. After observing the good rate capability performance up to 300 mAh g^−1^, the same cell was kept for extended cycling at a higher current rate. As shown in Figure 3e, the hybrid material showed an excellent cycling stability for 5000 cycles at a current density of 500 mA g^−1^. A specific discharge capacity value of 86 mAh g^−1^ was observed for the 5000th cycle, corresponding to a capacity retention value of 85%. A very stable Coulombic efficiency of ≈100% was maintained throughout the cycling (100% at 5000th cycle). Table S5, Supporting Information, shows a comparison of the electrochemical performance of structurally related electrode materials.^[^ ^42^, ^43^, ^59^, ^60^, ^61^, ^62^ ^]^ While the observed specific capacity fell into the typical range of 80–150 mAh g^−1^, **Melem‐PDI‐CNT** showed outstanding battery performance in terms of cyclability.

The electrochemical impedance spectra of both materials are shown in Figure 3f. Notably, the **Melem‐PDI‐CNT** hybrid electrode exhibits a remarkably lower charge transfer resistance compared to the pristine **Melem‐PDI** electrode. This pronounced decrease in charge transfer resistance indicates improved interfacial charge transport kinetics, which is consistent with the enhanced electrochemical performance observed for **Melem‐PDI‐CNT**. The improved conductivity is attributed to the incorporation of **CNTs**, which provide an interconnected conductive network that facilitates more efficient electron mobility at the electrode–electrolyte interface. This conductive matrix likely facilitates efficient electron transport and reduces resistive losses, contributing to the enhanced rate capability and overall electrochemical performance of the hybrid electrode, despite the absence of significant morphological or porosity differences, as confirmed by SEM and physisorption analyses.

### 2.3 Magnesium Battery Performance

**Figure 4:**
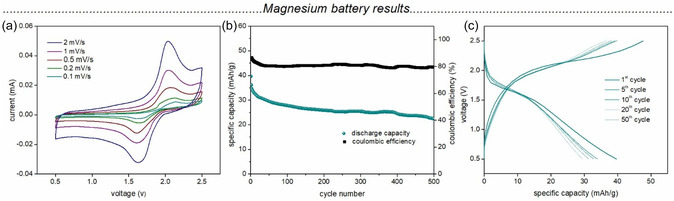
a) CV of **Melem‐PDI‐CNT** against Mg at various scan rates, b) battery cycling performance of **Melem‐PDI‐CNT** | Mg cell at a current rate of 200 mA g^−1^, c) galvanostatic charge–discharge profile of **Melem‐PDI‐CNT** | Mg cell for selected cycles.

The very stable cycling performance of the **Melem‐PDI‐CNT** hybrid material electrode for 5000 cycles at 500 mA g^−1^ in the Li half‐cells encouraged us to study the battery performance of the hybrid material in RMB. We chose the magnesium system due to its high potential for next‐generation batteries. Additionally, the advantageous reversible redox mechanism of the organic electrodes has potential for improved performance with the divalent Mg^2^^+^ ions. Because of the superior battery performance of the **Melem‐PDI‐CNT** cathodes over the **Melem‐PDI** cathodes in the Li‐ion cells, we decided to focus on the hybrid material for the Mg‐ion batteries. For the Mg‐ion half cells, 0.4 M solution of Mg[B(hfip)_4_]_2_ (magnesium tetrakis(hexafluoroisopropyloxy) borate) in 1,2‐dimethoxyethane (DME) was used as the electrolyte, whereas metallic Mg disks were used as counter electrodes (details in Supporting Information).^[^ ^63^, ^64^ ^]^ **Figure** **4**a represents the CV of the **Melem‐PDI‐CNT** hybrid material within the potential window of 0.5 − 2.5 V versus Mg/Mg^2+^ at various scan rates. In contrast to the Li half cells, which exhibit broad oxidation and reduction peaks, very prominent and sharp reversible redox peaks were observed in case of the Mg cell.

The reduction and oxidation peaks are centered around 1.63 and 2.03 V, respectively. Figure S21, Supporting Information, shows the CV of **Melem‐PDI** and **MWCNT** electrodes against Mg at a scan rate of 0.1 mVs^−1^. The CV profile of **Melem‐PDI** electrode shows redox peaks similar to those of **Melem‐PDI‐CNT,** confirming that the **Melem‐PDI** part in the hybrid material is responsible for the storage of Mg‐ions. The CV profile of **MWCNT** electrodes, on the other hand, does not show any redox activity in the given voltage window. The log–log plot of the peak current (*i*) versus the scan rate (*υ*) obtained from the CV at various scan rate is presented in Figure S22, Supporting Information. The b values for the cathodic and anodic scans were found to be 0.54 and 0.8 indicating a diffusion‐controlled mechanism of the interaction of the Mg‐ion with the organic framework. Figure 4b represents the battery cycling performance of the hybrid material electrode at a current rate of 200 mA g^−1^ for 500 cycles. The initial specific discharge capacity of 40 mAh g^−1^ has a 58% retention value after 500 cycles. Based on the well‐defined reduction and oxidation peaks observed in the CV plot of the Mg systems, along with the low specific capacity, it can be hypothesized that only roughly half of the carbonyl groups in the **Melem‐PDI** material are participating in the redox process. Table S6, Supporting Information, provides a literature comparison with previously reported organic magnesium battery results, suggesting overall a comparable battery performance of the **Melem‐PDI‐CNT** material.^[^ ^37^, ^65^, ^66^ ^]^ The initial Coulombic efficiency of 86% shows a slight decrease to 81% after the 30th cycle and remains almost constant thereafter. Figure 4c shows the galvanostatic charge–discharge profile for selected cycles, which indicates relatively flat charge and discharge plateaus, when compared to the Li‐ion batteries based on the same organic electrode material. The battery cycling performance of the **Melem‐PDI** electrode at 200 mA g^−1^ for 50 cycles is shown in Figure S23, Supporting Information, for comparison. Similar reduction and oxidation plateaus as that of the **Melem‐PDI‐CNT** further confirms the **Melem‐PDI** part in the hybrid material to be the redox‐active component. A significantly lower capacity values were observed in the case of **Melem‐PDI** (4.58 mAh g^−1^ for 1st cycle) as compared to the **Melem‐PDI‐CNT** electrodes. Thus, it can be concluded that although the MWCNTs show no redox behavior (as seen in CV) in the selected voltage range, they improve the battery performance by increasing the electronic conductivity and surface area of the hybrid material to provide better accessibility for the Mg^2+^ ions.

## 3 Conclusion

In conclusion we have synthesized a new PDI‐based polymeric network material containing PTCDA and heptazine unit (**Melem‐PDI**) using a simple one‐step condensation reaction. Further in situ incorporation of MWCNT yielded a hybrid material **Melem‐PDI‐CNT** with enhanced electronic conductivity. **Melem‐PDI‐CNT** exhibited excellent cycling stability as positive electrode for LIB at a current rate as high as 500 mA g^−1^ for 5000 cycles. The hybrid material further demonstrated promising performance as positive electrode material in magnesium batteries. The results indicated reversible interaction of the Mg^2+^ ions with the functional groups integrated in the polymeric network, giving rise to a stable battery cycling for 500 cycles at 200 mA g^−1^. Kinetic study revealed a predominant pseudocapacitive storage mechanism for the Li^+^ ions, whereas a diffusion‐controlled mechanism was observed in case of Mg^2+^ ions. We expect that this study will prove useful for the design of further positive electrode active materials. Future work will focus on clarifying the role of residual zinc^[^ ^67^ ^]^ and the creation of all‐organic batteries^[^ ^10^ ^]^ since a chemically related composite was recently demonstrated as negative electrode active material.^[^ ^52^ ^]^

## Conflict of Interest

The authors declare no conflict of interest.

## Supporting information

Supplementary Material

## Data Availability

The data that support the findings of this study are openly available in Zenodo at https://zenodo.org/uploads/16994733, reference number 16994733.
