# Supplementary material for: Melem‐Perylene Diimide Polymer Network as Efficient Positive Electrode for Rechargeable Lithium and Magnesium Batteries
Source: ChemSusChem. 2025 Sep 8;18(20):e202500967. doi: 10.1002/cssc.202500967 (PMC12548948; doi:10.1002/cssc.202500967)
Supplement: Supplementary file 1 — Supplementary Material [file CSSC-18-e202500967-s001.pdf]

Supporting Information
©Wiley-VCH 2021
69451 Weinheim, Germany

Melem-PDI polymer network as efficient positive electrode for rechargeable lithium and magnesium batteries

Ruth Gomes,[a] Jan Kraus,[a] Igor Krivtsov,[b,c] Vivek Wakchaure,[a] Sibylle Riedel,[d] Zhirong Zhao-Karger,[d,e] Johannes Liessem[c] Christof Neumann,[f] Martin Oschatz,[g,h] Andrey Turchanin,[f] Maximilian Fichtner,[d,e] Radim Beranek,[c] Max von Delius*[a]

[a] Dr. R. Gomes, J. Kraus, Dr. V. Wakchaure, Prof. Dr. M. von Delius
Institute of Organic Chemistry, Ulm University
Albert-Einstein-Allee 11, 89081 Ulm (Germany)
E-mail: max.vondelius@uni-ulm.de

[b] Dr. I. Krivtsov
Department of Chemical and Environmental Engineering, University of Oviedo
Avenida Julián Clavería 8, 33006 Oviedo (Spain)

[c] Dr. I. Krivtsov, J. Liessem, Prof. Dr. R. Beranek
Institute of Electrochemistry, Ulm University
Albert-Einstein-Allee 47, 89081 Ulm (Germany)
E-mail: radim.beranek@uni-ulm.de

[d] Dr. S. Riedel, Dr. Z. Zhao Karger, Prof. Dr. M. Fichtner
Helmholtz Institute Ulm (HIU), Electrochemical Energy Storage,
Helmholtzstraße 11, 89081 Ulm (Germany)

[e] Dr. Z. Zhao Karger, Prof. Dr. M. Fichtner
Institute of Nanotechnology, Karlsruhe Institute of Technology,
Hermann-von-Helmholtz-Platz 1, 76344 Eggenstein-Leopoldshafen (Germany)

[f] Dr. C. Neumann Prof. Dr. A. Turchanin
Institute of Physical Chemistry, Friedrich-Schiller University Jena
Lessingstraße 10, 07743 Jena, Germany

[g] Prof. Dr. Martin Oschatz
Institute for Technical Chemistry and Environmental Chemistry, Friedrich Schiller University Jena
Philosophenweg 7a, 07743 Jena (Germany)

[h] Prof. Dr. Martin Oschatz
Helmholtz Institute for Polymers in Energy Applications Jena (HIPOLE Jena)
Lessingstraße 12–14, 07743 Jena (Germany)

Table of Contents

General Experimental Information 3
Synthesis and characterization data 5
Synthesis of Melem 5
Synthesis of Melem-PDI material 6
Synthesis of Melem-PDI-CNT material 6
NMR spectra 7
XPS Data 13
SAXS Data 17
TGA Data 18
UV-Vis Spectra 19
SEM Data 20
TEM Data 21
Electrochemical characterization and battery testing 22
Calculation of theoretical specific capacity 23
Cyclic voltammograms for Li battery 25
Electrochemical kinetic experiment for Li battery 25
Post cycling FT-IR analysis on electrode 26
Cyclic voltammograms for Mg battery 27
Electrochemical kinetic experiment for Mg battery 27
Battery cycling of Melem-PDI | Mg cell 28
Summary of elemental and XPS analysis 29
Literature comparison 30
References 32

General Experimental Information

All the solvents and starting materials used for synthesis were obtained from commercial sources: Sigma Aldrich (Schnelldorf, Germany), Alfa Aesar GmbH & Co KG (Karlsruhe, Germany), and TCI (Tokyo Chemical Industry Co. Ltd., Tokyo, Japan). Multi-walled carbon nanotubes (as-produced cathode deposit, >7.5% MWCNT basis, O.D. × L 7-15 nm × 0.5-10 µm, avg. no. of layers, 5 - 20) were obtained from Sigma Aldrich (Schnelldorf, Germany).

All solid-state NMR experiments were conducted using a Bruker AVANCE NEO 400 spectrometer with a double resonance (H/X) CPMAS-probe head. Standard ZrO_2 rotors with 4 mm diameter were used for solid samples. ^{13}C shifts were referenced to Adamantane, ^{15}N shifts to $^{15}NH_4Cl$. ^{13}C NMR spectra were recorded at 14 kHz spinning frequency using a SPINAL-64 sequence for broadband proton decoupling, while ^{15}N spectra were recorded at 4 kHz using an H-X-CP pulse program with SWf-TPPM decoupling sequence.

FT-IR spectroscopic data was acquired on an ALPHA II FTIR (Bruker Optics, Germany) instrument.

UV-Vis absorption spectra were recorded using a Perkin Elmer Lambda 365 spectrometer.

For Elemental analysis a vario EL cube and vario MICRO cube (Elementar Analysensysteme GmbH, Langenselbold, Germany) were used.

For thermogravimetric analysis a TGA 8000 TM from Perkin Elmer (PerkinElmer Inc., Waltham, Massachusetts, USA) with PYRIS-software V. 13 was used, samples were pyrolized in Alox-pans under N_2 atmosphere.

The specific surface areas (SSA), pore volumes of the synthesized materials were determined from N_2 adsorption-desorption isotherms registered using a Micromeritics ASAP2020 system. Before analysis, the samples were degassed under vacuum at 150 °C for 4 h, then the measurement was performed at liquid nitrogen temperature (-196 °C). The Brunauer-Emmett–Teller (BET) method was used to calculate the SSA.

Powder X-ray diffraction (PXRD) measurements are performed via Panalytical X´Pert MPD Pro, Bragg-Brentano, reflection, Cu-Ka, X´Cellerator.

The scanning electron microscopic (SEM) images were recorded on a Hitachi S-5200 cryo-scanning electron microscope.

The transmission electron microscopic images were obtained using Jeol 1400 transmission electron microscope at 120 kV.

The XPS data was measured using a K-Alpha X-ray Photoelectron Spectrometer System (Thermo Fisher Scientific) with a monochromatic X-ray source (Al K_α) with a spot diameter of 400 μm and an electron detector with 0.5 eV energy resolution. A flood gun was employed for charge compensation. The spectra were calibrated using the C 1s peak (284.6 eV) and fitted using Voigt functions after background subtraction.

Synthesis and characterization data

Synthesis of Melem

Melem was prepared by a modified procedure reported by Shalom *et al.* [10.1039/D1NR06974H]. For this, 5 g of dicyandiamide was placed in a lid-covered ceramic crucible and heated in a muffle furnace with a rate of 5 °C/min till the temperature reached 425 °C that was maintained for 4 h. After the thermal condensation, the synthesized melem was left to cool down to room temperature, and the obtained powder was repeatedly washed by centrifugation with deionized water.

^{13}C CP MAS NMR (101 MHz, 25 °C): δ =164.28, 154.10 ppm.

^{15}N CP MAS NMR (25 °C): δ = 176.74 (aromatic N), 107.87 (NH_2) ppm.

IR: 3460, 3265, 3073, 1595, 1440, 1400, 1290, 1086, 790 cm^{-1}.

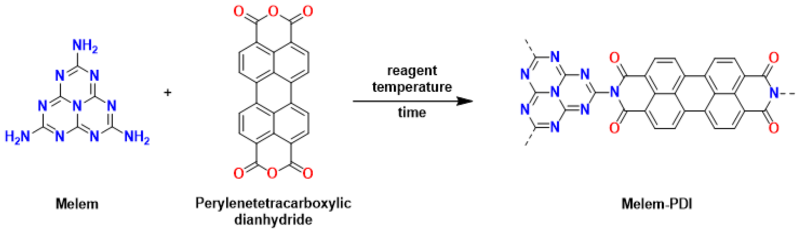

Entry	Reagent	Temperature	Time (day)	Observation
1	$ZnSO_4$, pyridine	150	1	No polymerization
2	$ZnSO_4$, H_2SO_4	200	1	No polymerization
3	$ZnCl_2$	300	2	Partial decomposition (59% black solid isolated)
4	$ZnCl_2$, KCl, NaCl	250	3	Successful polymerization (85% dark purple solid isolated)

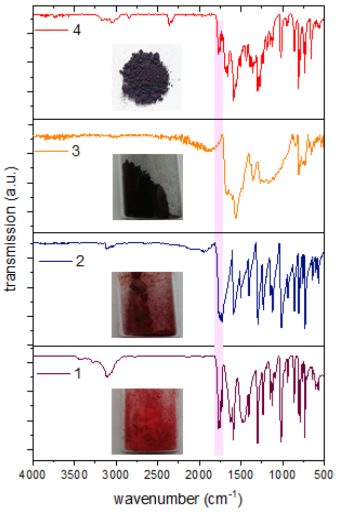

Scheme S1: Synthesis of conditions tested for the **Melem-PDI** material and corresponding FTIR.

Note: Entries 1 and 2 showed no evidence for polymer formation. FTIR spectra of isolated solids (38% for 1 and 17% for 2) show starting anhydride and no imide linkage formation was observed. Entry 3 was judged to be promising, however, the elevated temperature leads to partial decomposition of the starting material, resulting in reduced yield (59%) and unspecific features in the FTIR spectrum. Entry 4, employing ionothermal synthesis with a three-salt eutectic mixture, appeared particularly effective in promoting polymerization (85%).

Synthesis of Melem-PDI material

Zinc (II) chloride (5.31 g), potassium chloride (1.85 g) and sodium chloride (0.54 g) were mixed and molten at 250 °C. The melt was stirred for 0.5 h *in vacuo* and subsequently cooled to room temperature. Perylenetetracarboxylic dianhydride (PTCDA) (272 mg, 0.69 mmol, 1.5 eq.) and Melem material (102 mg, 0.47 mmol, 1.0 eq.) were added to the prepared salt mixture and were ground together using a mortar and a pestle. Vacuum was applied (0.1 mbar) and the mixture was heated at 250 °C for 3 days. The deep purple mixture was cooled to room temperature and suspended in hydrochloric acid (2 M, 25 mL). The mixture was filtered through a PTFE filter (Whatman, 0.2 µm pore size), washed with hydrochloric acid (2 M, 25 mL), water (25 mL) and methanol (3 x 25 mL) and dried *in vacuo*.

298 mg (isolated yield 85 %) of a deep purple solid was obtained.

^{13}C CP MAS NMR (101 MHz, 25 °C): δ = 173.07, 163.61, 159.81, 134.61, 132.92, 131.47, 127.04, 122.91, 120.82, 117.13 ppm.

^{15}N CP MAS NMR (25 °C): δ = 164.37 (aromatic N) ppm.

IR: 1754, 1690, 1662, 1592, 1547, 1375, 1300, 1275, 1020, 858, 807, 735, 657 cm^{-1}.

UV/Vis (H_2SO_4, 95%): 552 nm, 594 nm

Synthesis of Melem-PDI-CNT material

Zinc (II) chloride (3.34 g), potassium chloride (1.18 g) and sodium chloride (0.34 g) were mixed and molten at 250 °C. The melt was stirred for 0.5 h *in vacuo* and subsequently cooled to room temperature. Perylenetetracarboxylic dianhydride (PTCDA) (178 mg, 0.45 mmol, 1.5 eq.), Melem material (66 mg, 0.30 mmol, 1.0 eq.) and MWCNTs (104 mg, 30% wt/wt with respect to PTCDA, Melem and MWCNT mixture) were mixed, ground with mortar and pestle and added to the previously prepared salt mixture. Vacuum was applied (0.1 mbar) and the mixture was heated to 250 °C for 4 d. The deep purple mixture was cooled to room temperature and suspended in hydrochloric acid (2 M, 30 mL). The mixture was filtered through a PTFE filter (Whatman, 0.2 µm pore size), washed with hydrochloric acid (2 M, 2 x 30 mL), water (deionized, 3 x 30 mL) and methanol (3 x 30 mL) and dried *in vacuo*.

308 mg (isolated yield 94 %) of a deep purple solid was obtained.

^{13}C CP MAS NMR (101 MHz, 25 °C): δ = 173.04, 163.7, 160.04, 134.75, 132.61, 131.54, 127.02, 122.62, 120.89, 117.25 ppm.

IR: 1754, 1690, 1662, 1592, 1547, 1440, 1375, 1300, 1275, 1020, 858, 807, 735, 657 cm^{-1}.

NMR spectra

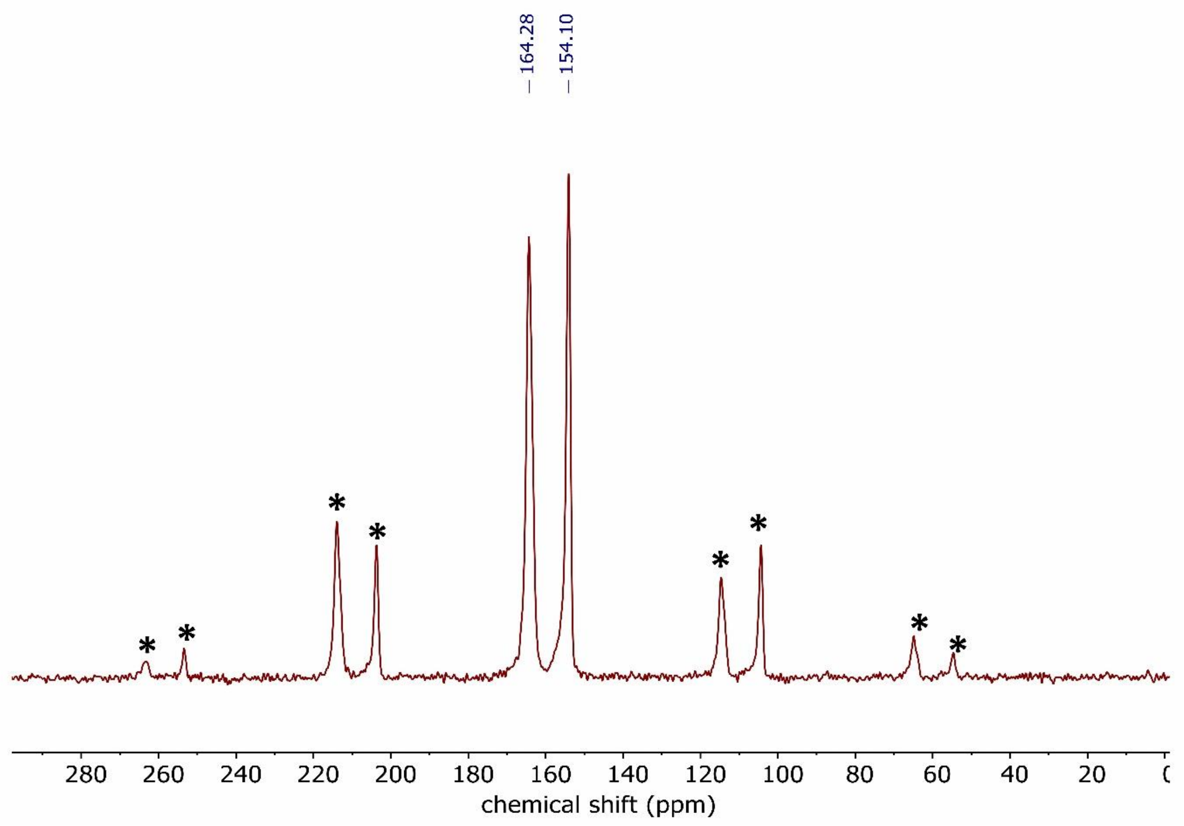

Figure S1. ^{13}C CP MAS NMR (101 MHz) spectrum of **Melem** (*'s indicates spinning sidebands).

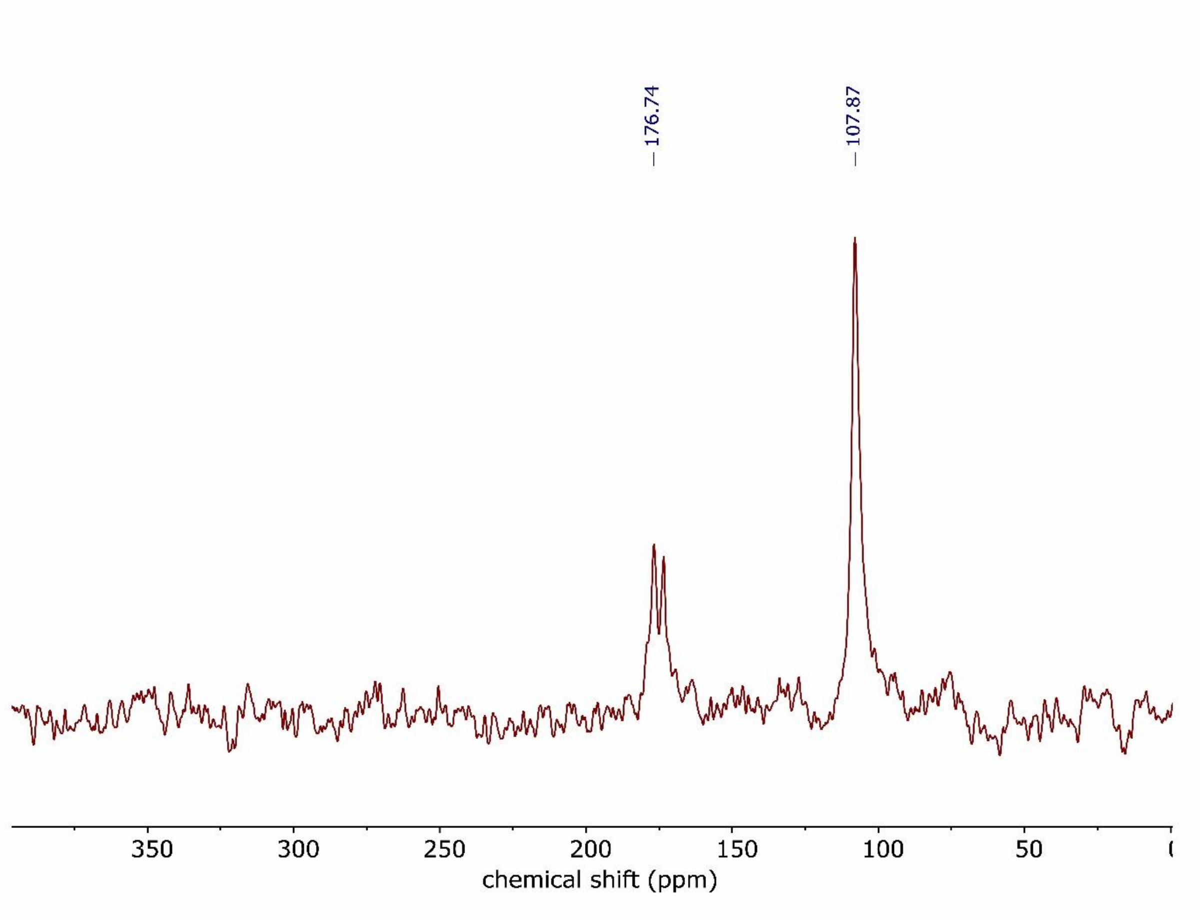

Figure S2. ^{15}N CP MAS NMR spectrum of **Melem**.

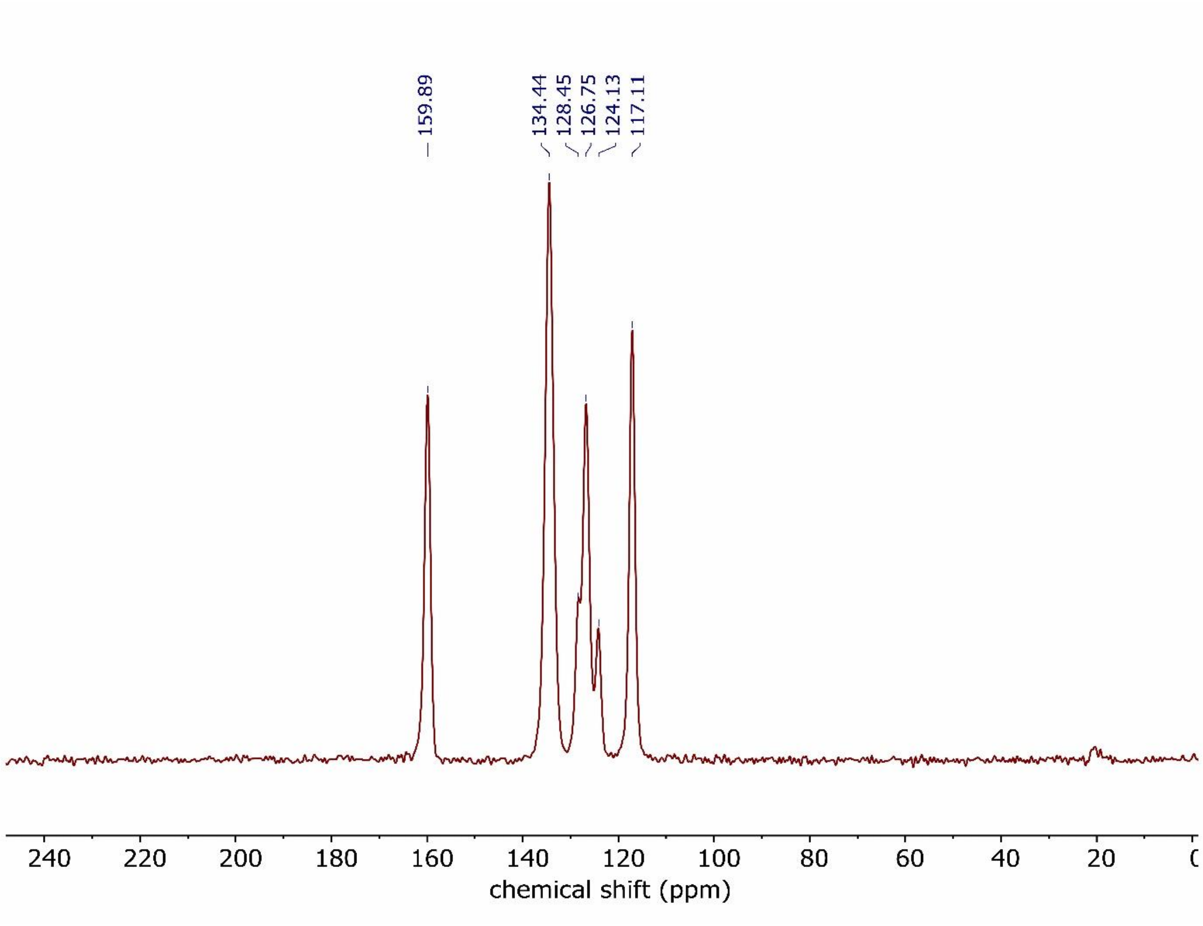

Figure S3. ^{13}C CP MAS NMR (101 MHz) spectrum of **PTCDA**.

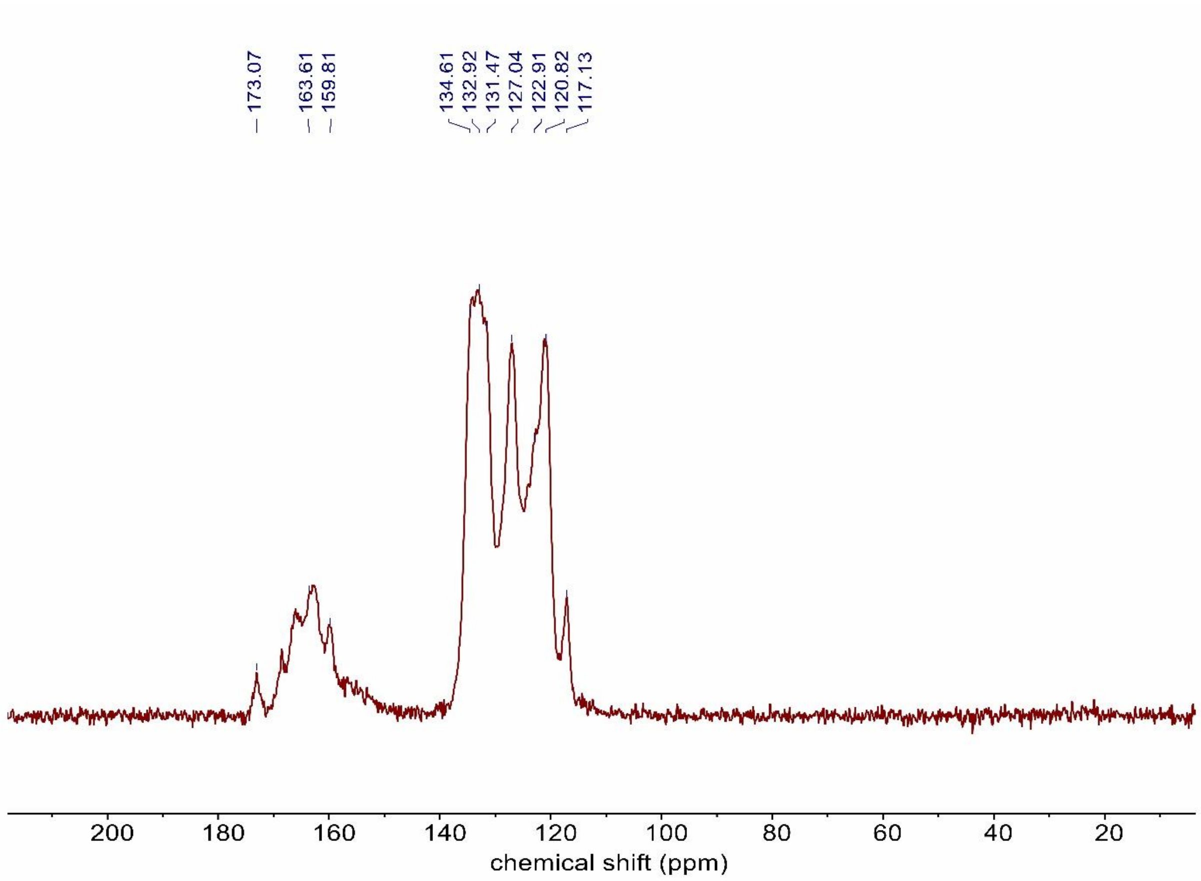

Figure S4. ^{13}C CP MAS (101 MHz) NMR spectrum of **Melem-PDI** material.

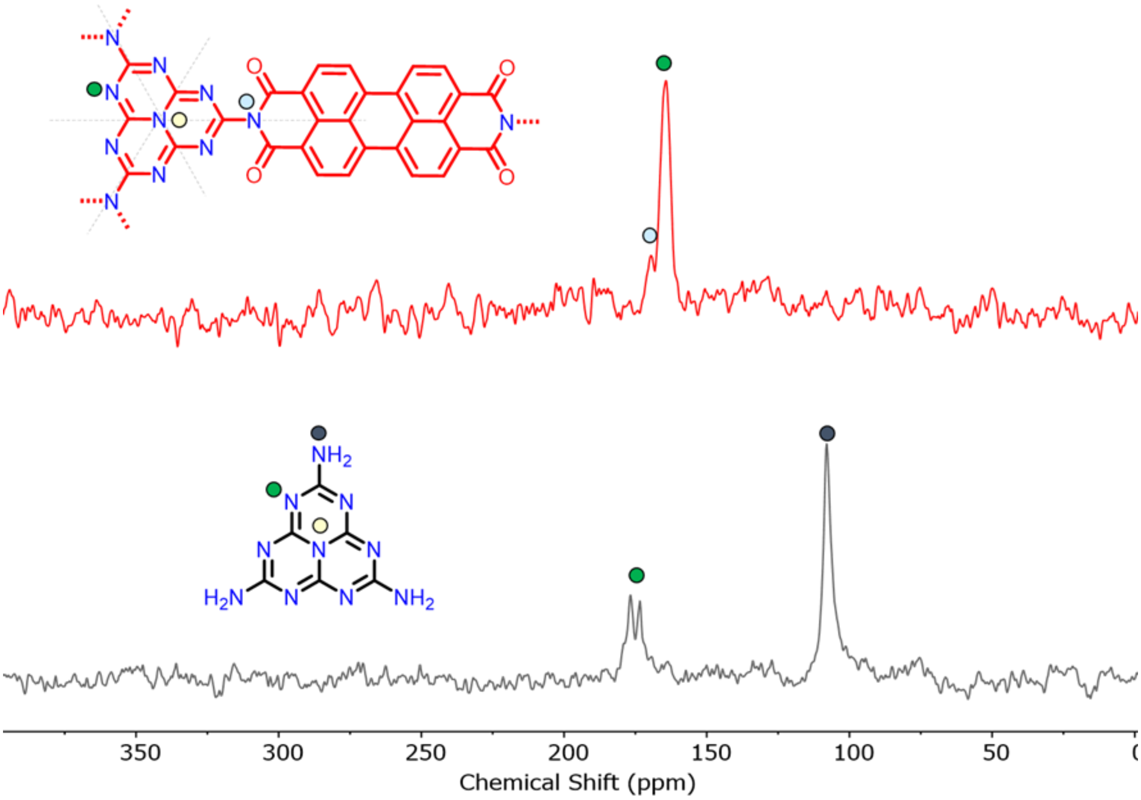

Figure S5. Stacked ^{15}N CP MAS NMR spectrum of **Melem** and **Melem-PDI**.

The signals were referenced to ammonium chloride (15N). The spectrum was recorded on a sample with natural isotopic abundance, not ^{15}N-enriched material. Note: Center N atom (Nc) was not observed due to several factors; owing to its tertiary nature, Nc is less effectively cross-polarized due to the lack of direct bonding to hydrogen atoms. It also tends to have a shorter T1 relaxation time and exists in a rigid, highly symmetric environment, which enhances quadrupolar interactions. These combined factors lead to rapid relaxation and significant line broadening, making the signal broader and weaker than those of the peripheral nitrogen atoms. As a result, under the experimental conditions employed in our study, the Nc signal remains undetectable.

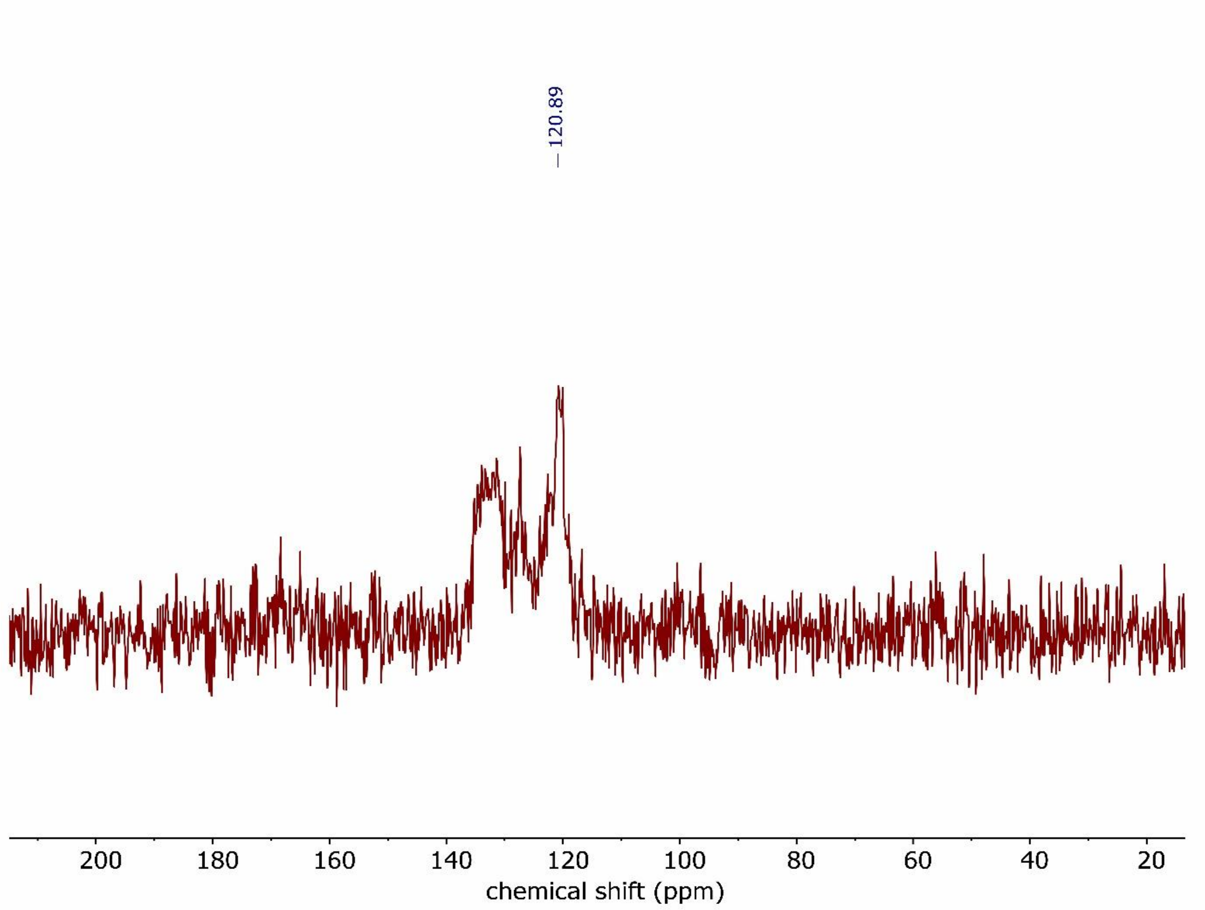

Figure S6. ^{13}C CP MAS NMR (101 MHz) spectrum of **Melem-PDI-CNT** material.

XPS Data

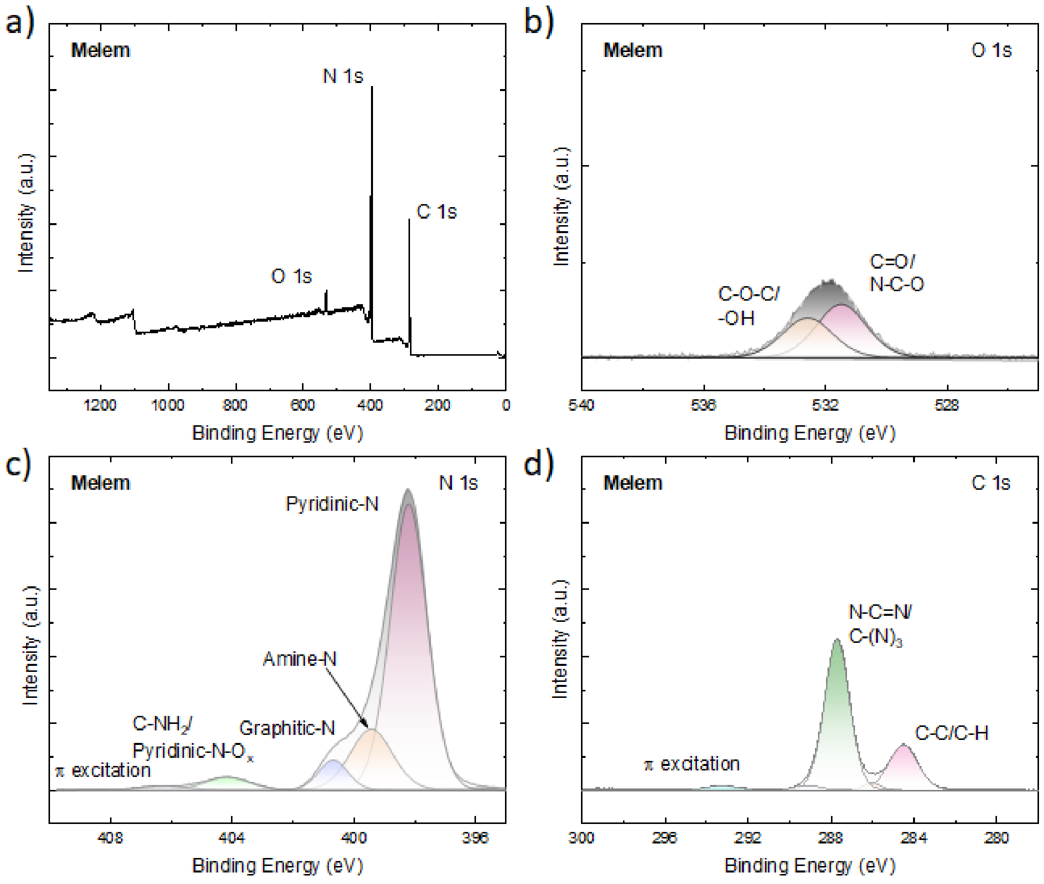

Figure S7. a) XP survey and High-resolution b) O 1s, c) N 1s, and d) C 1s XP spectra obtained for the **Melem**.

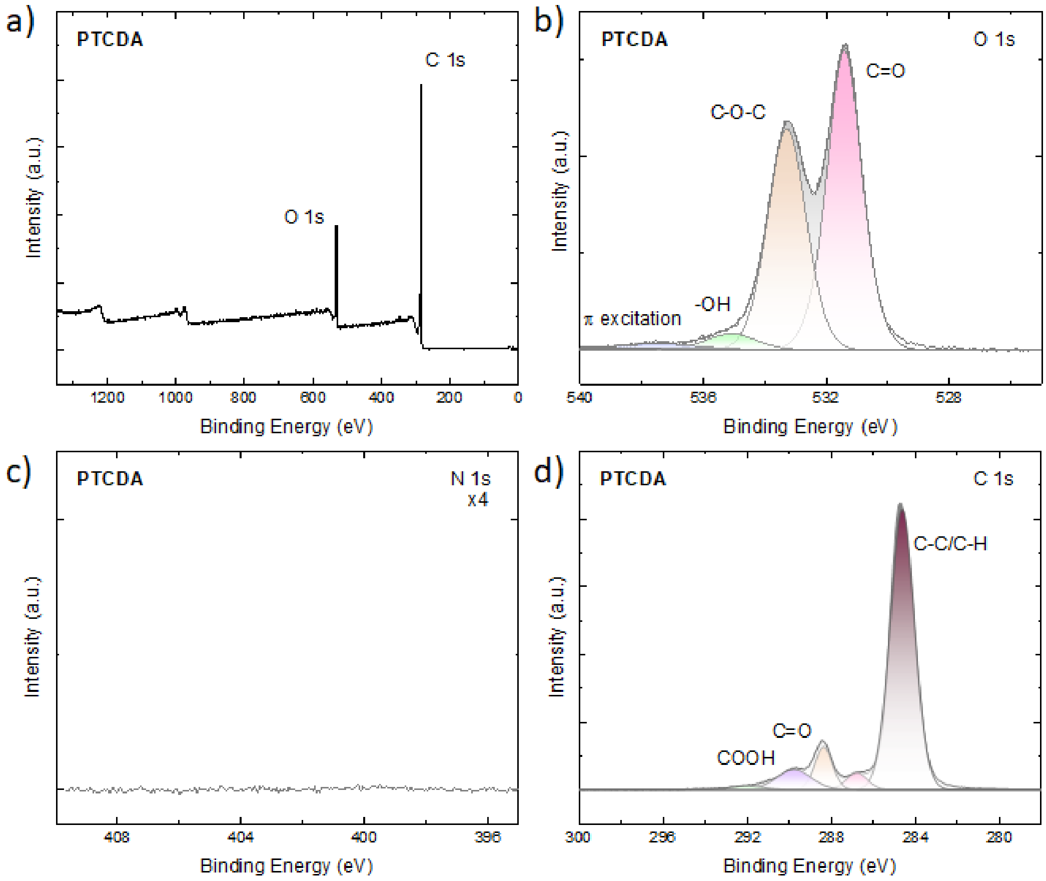

Figure S8. a) XP survey and High-resolution b) O 1s, c) N 1s, and d) C 1s XP spectra obtained for the **PTCDA**. For better visibility, the N 1s spectrum is multiplied by a factor of 4 with respect to the N 1s spectrum in Figure S7.

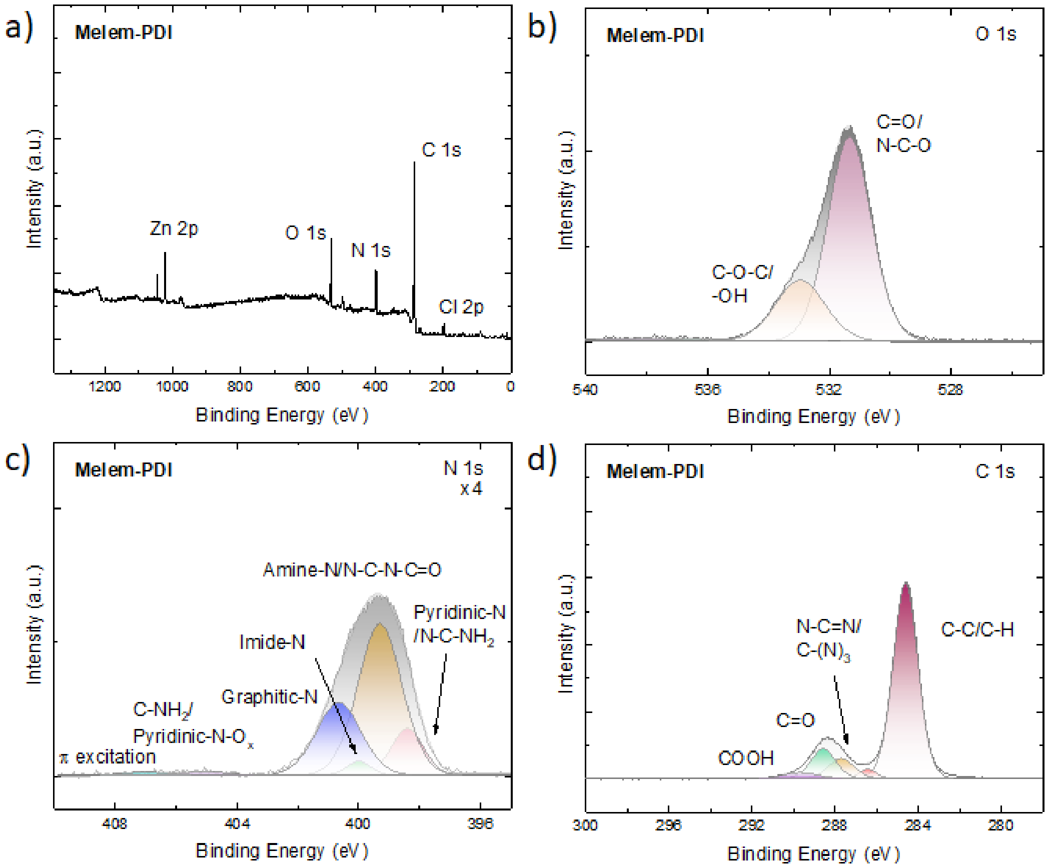

Figure S9. a) XP survey and High-resolution b) O 1s, c) N 1s, and d) C 1s XP spectra obtained for the **Melem-PDI**. For better visibility, the N 1s spectrum is multiplied by a factor of 4 with respect to the N 1s spectrum in Figure S7.

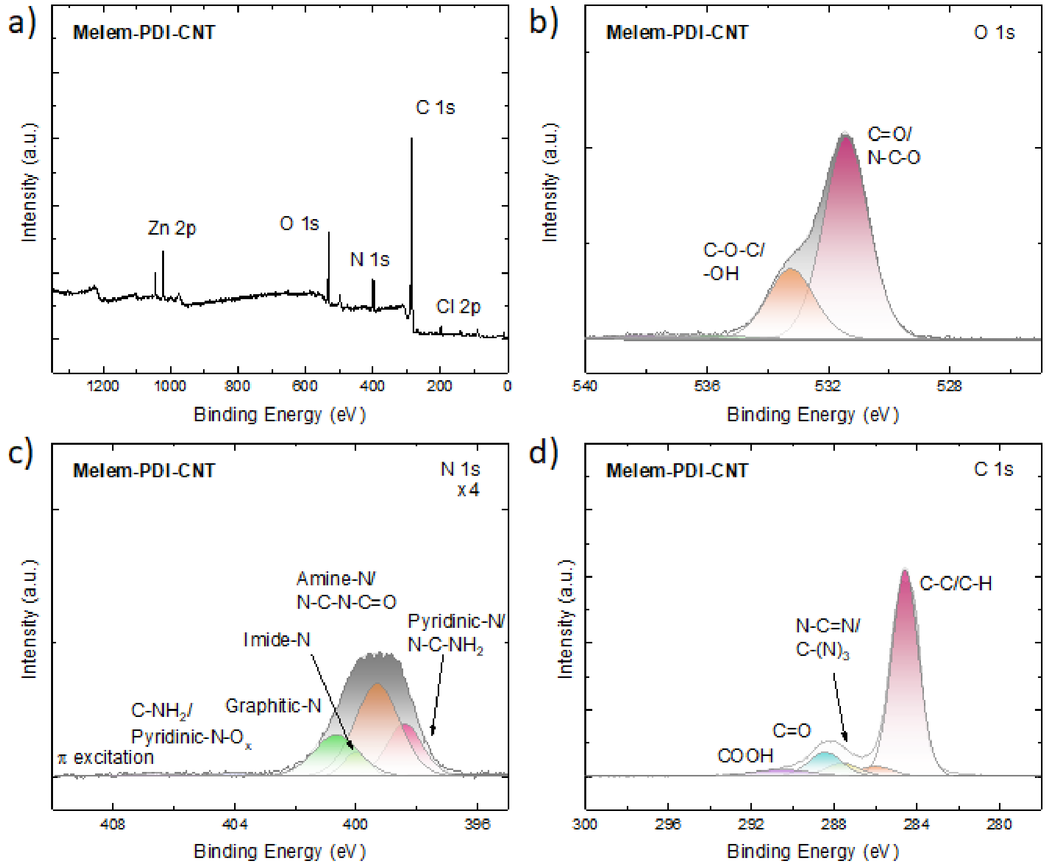

Figure S10. a) XP survey and High-resolution b) O 1s, c) N 1s, and d) C 1s XP spectra obtained for the **Melem-PDI-CNT**. For better visibility, the N 1s spectrum is multiplied by a factor of 4 with respect to the N 1s spectrum in Figure S7.

SAXS Data

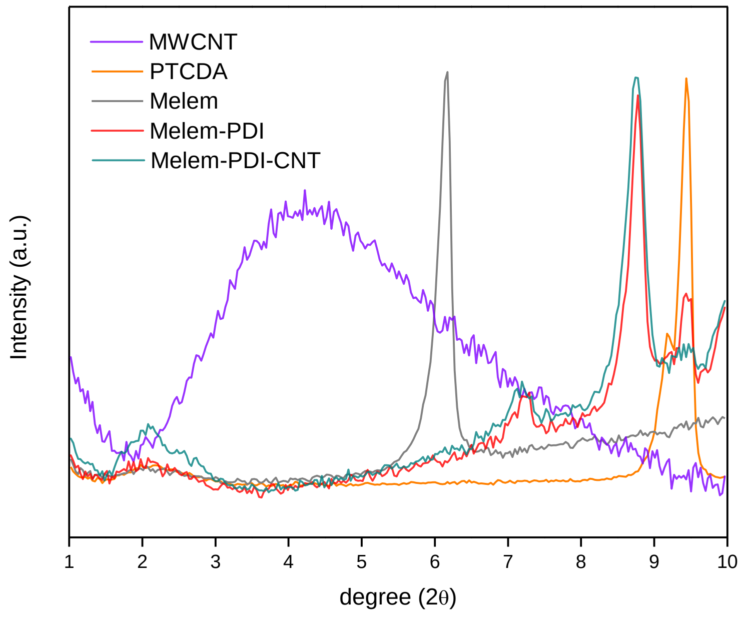

Figure S11. Small-angle X-ray scattering (SAXS) for both materials and the precursors.

TGA Data

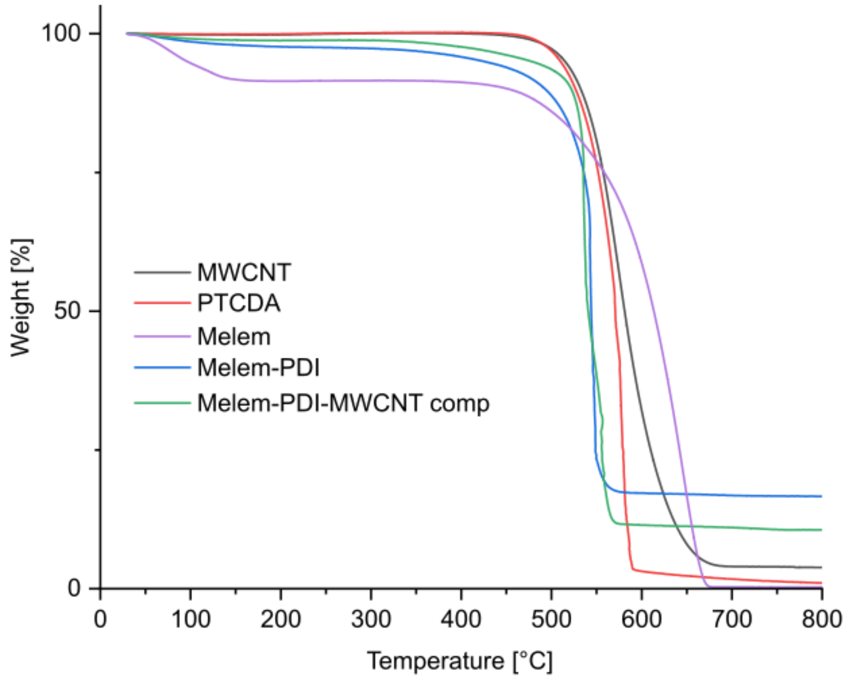

Figure S12. Thermogravimetric weight loss profile of both materials and the precursors recorded under oxygen atmosphere.

UV-Vis Spectra

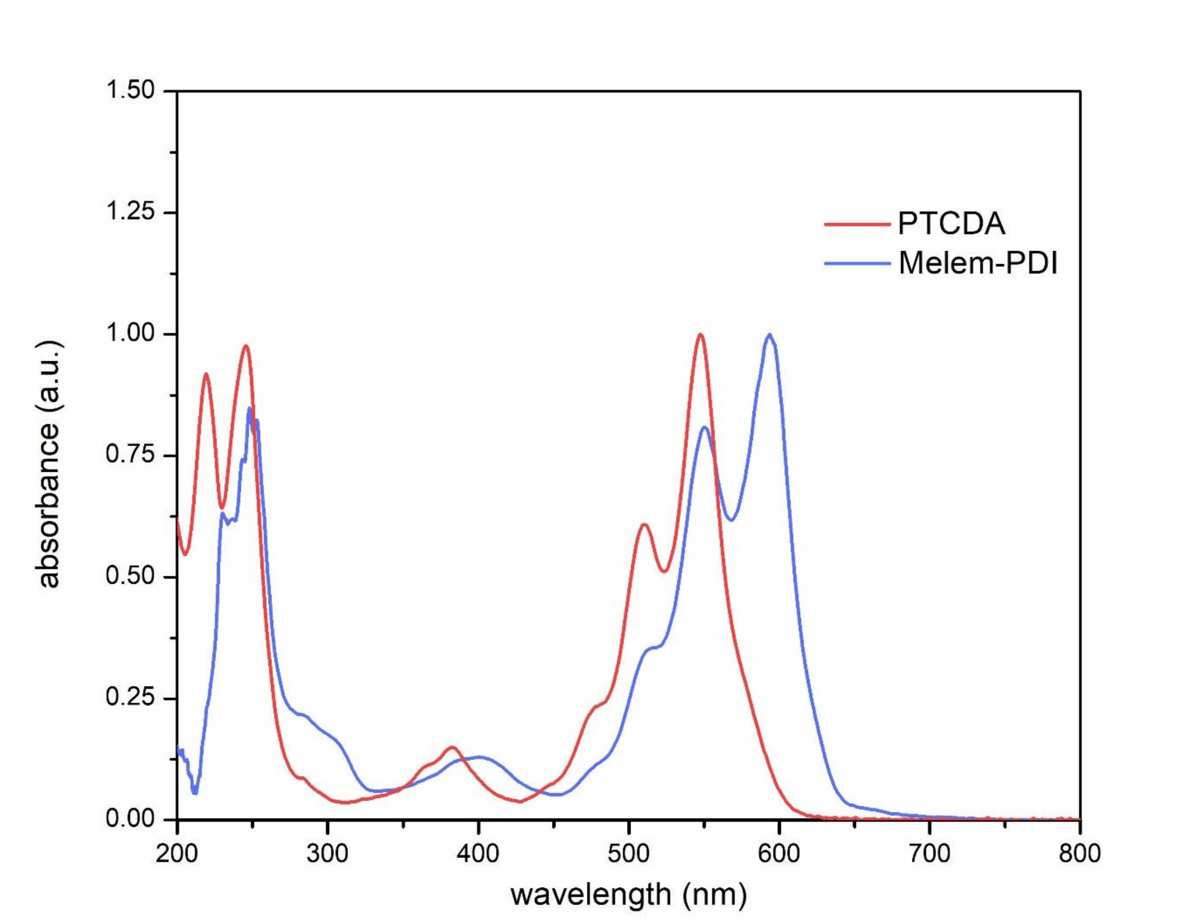

Figure S13. UV-Vis spectra of **Melem-PDI** and **PTCDA** material in conc. H_2SO_4

SEM Data

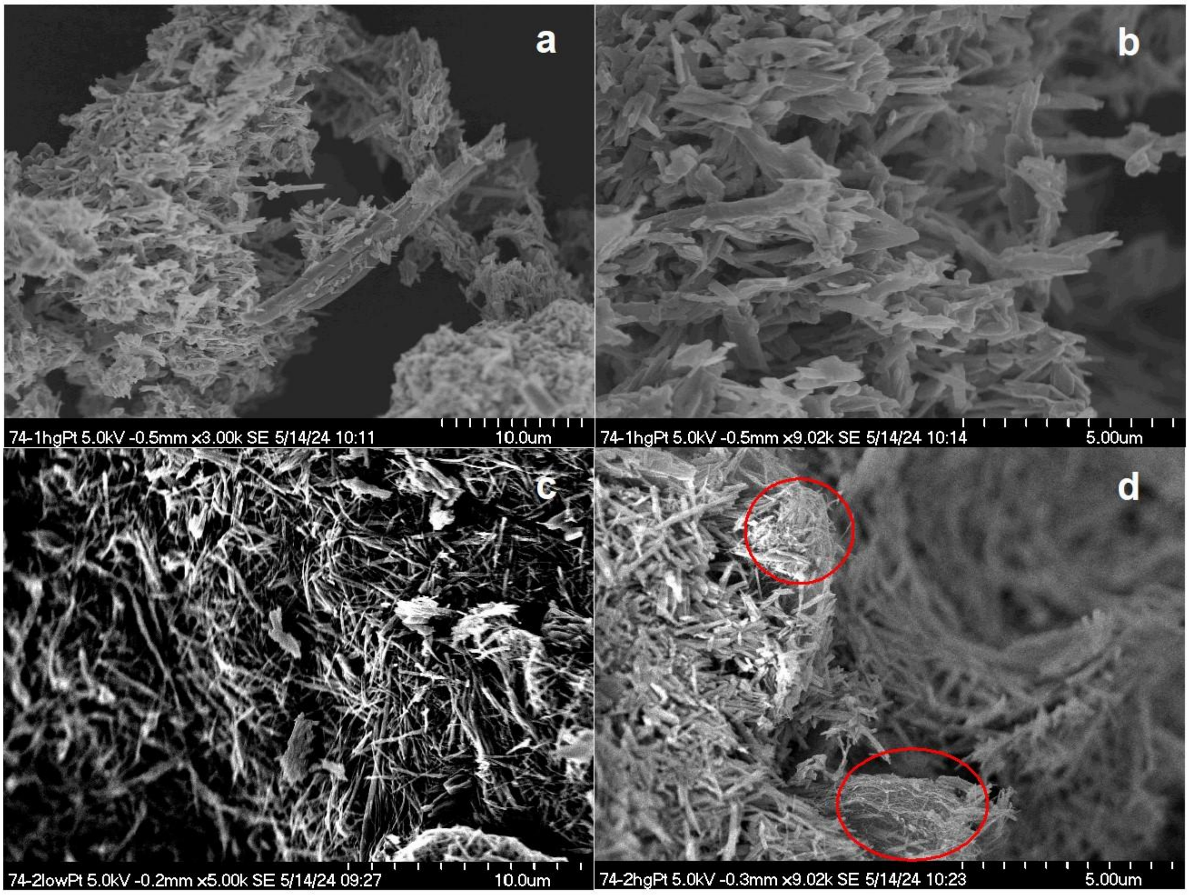

Figure S14: Scanning electron microscopic image of (a, b) **Melem-PDI** and (c, d) **Melem-PDI-CNT**. Clusters of carbon nanotubes can be observed within the red circles for **Melem-PDI-CNT**.

TEM Data

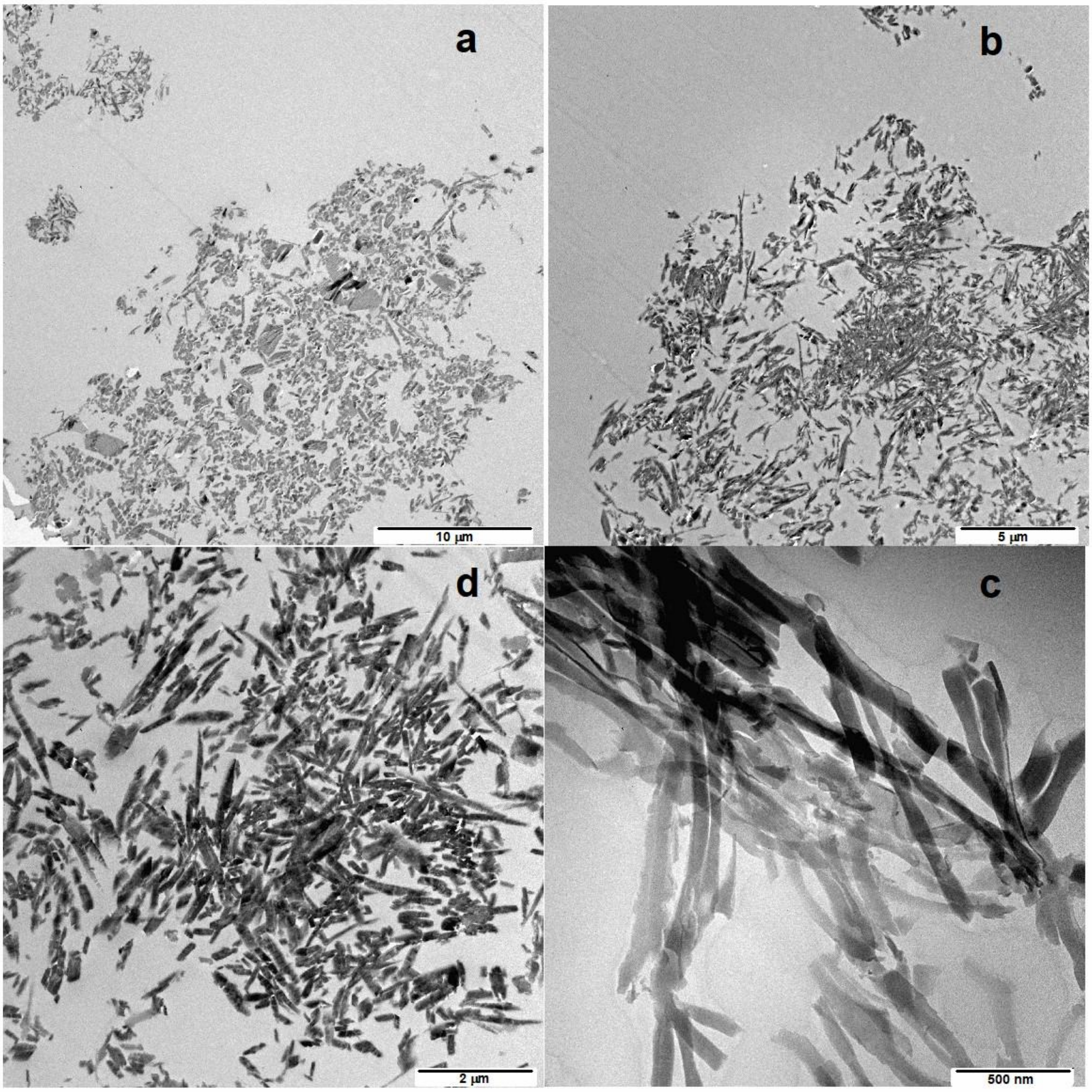

Figure S15: Transmission electron microscopic image of **Melem-PDI** material at various depth of magnification.

Electrochemical characterization and battery testing

All the solvents and salts required for the electrolyte preparation and electrode casting and Li-foil (thickness 0.6 mm, purity 99.9 %) and Mg-disks (thickness 0.25 mm, 10 mm diameter, purity 99.9%) for battery assembly were obtained in pure form (battery grade) from Sigma Aldrich and transferred into an argon-filled glovebox immediately. The salts were dried in a glass oven (Büchi, B-585) under vacuum at 80 ºC for 12 h before use and the solvents were stored over molecular sieves. The coin cells (type 2032) were purchased from PI-KEM (Tamworth, UK).

The electrodes were prepared using a slurry containing 7:2:1 weight ratio of **Melem-PDI**/**Melem-PDI-CNT**, acetylene black and PVDF in *N*-methyl-2-pyrrolidone (NMP) solvent. For the bare MWCNT electrodes, an electrode composition of 2.1:6.9:1 (MWCNT: Carbon Black: PVDF) was used to maintain a similar percentage of MWCNT as in the case of **Melem-PDI-CNT** electrode. Carbon coated aluminum foil was used as the current collector and the coating was done using a doctor blade and dried in a vacuum oven for 12 h at 80 ºC. 12 mm circular disks of electrodes were punched out and dried in glass oven (Büchi, B-585) under vacuum at 80 ºC for 12 h before transferring to the glovebox. The active material loading of the **Melem-PDI** and **Melem-PDI-CNT** electrodes was maintained to be ~0.6 mg/cm^2 whereas in case of the MWCNT electrodes the loading was ~ 0.2 mg cm^{-2} . For testing against lithium, 12 mm circular disks of lithium foil were cut and used as the anode. 1 M LiTFSI + 0.25 M $LiNO_3$ in 1:1 (V:V) mixture of dioxolane (DOL) and 1,2-dimethoxyethane (DME) was used as the electrolyte (120 µL electrolyte per cell). For the Mg half cells Mg-disks (10 mm) were used as the anode and 0.4 M $Mg[B(hfip)_4]_2{\cdot}3$ DME in DME (120 µL per cell) as the electrolyte. All the batteries were assembled in coin cells type 2032 using Whatman glass fiber GF/D as separator (16 mm) in an argon filled glovebox with oxygen and water level less than 0.1 ppm.

Cyclic voltammograms, galvanostatic charge discharge cycling and electrochemical impedance spectra were recorded with a BCS-805 battery cycler from Biologic (Seyssinet-Pariset, France).

The "the raw files for the battery data " can be downloaded from the Zenodo repository using the following link: https://doi.org/10.5281/zenodo.15372995.

Calculation of theoretical specific capacity

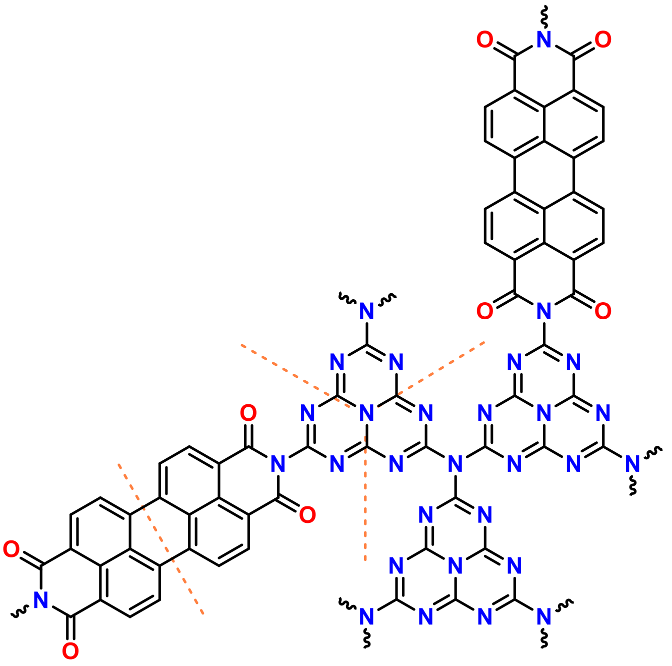

Molecular weight of idealised repeating unit = 251.21 $gmol^{-1}$

Figure S16: Structure of the Melem-PDI polymeric material showing the idealise repeating units.

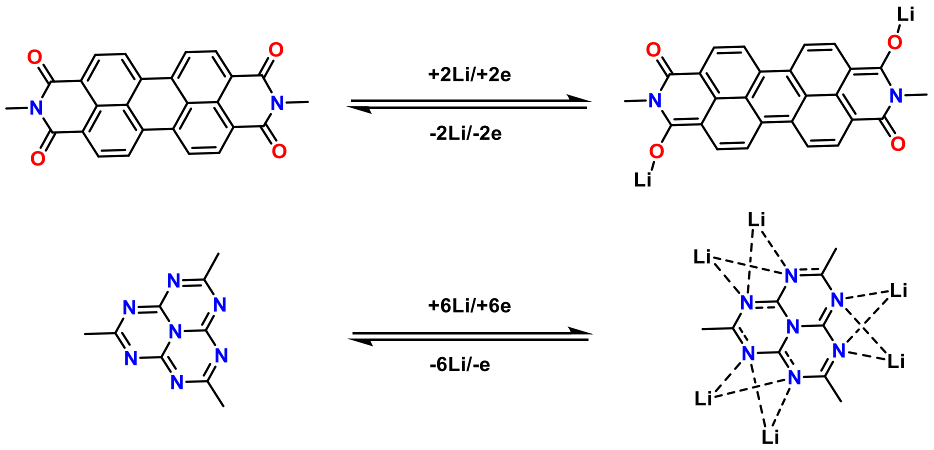

Figure S17: Plausible reaction mechanism for the redox reaction occurring per unit of PDI and Melem molecules.

Each repeating unit consist of one-third of melem unit and half of PDI unit

Therefore, molecular weight of each repeating unit

$$= 1/3 \text{ x } 171.12 + 1/2 \text{ x } 388.34 \text{ g mol}^{-1}$$

$= 251.21 \text{ g mol}^{-1}$

The theoretical specific capacity (C_t) of the **Melem-PDI** polymeric network, considering each repeating unit contribute to a 3-electron (1e$^-$ from half PDI and 2e$^-$ from 1/3 Melem unit) unit redox process:

$$C_t = \frac{nF\ (C * mol^{-1})}{mol\ wt\ (g * mol^{-1})}$$

$$= \frac{3 * 96500\ C}{251.21\ g}$$

$$= \frac{3 * 96500\ mAh}{251.21\ g * 3.6}$$

$$= 320.12\ \text{mAhg}^{-1}$$

The theoretical specific capacity (C_t) of the **Melem-PDI** polymeric network, considering each repeating unit contribute to a 2-electron (1e$^-$ from half PDI and 1e$^-$ from 1/3 Melem unit) unit redox process:

$$C_t = \frac{nF\ (C * mol^{-1})}{mol\ wt\ (g * mol^{-1})}$$

$$= \frac{2 * 96500\ C}{251.21\ g}$$

$$= \frac{2 * 96500\ mAh}{251.21\ g * 3.6}$$

$$= 213.41\ \text{mAhg}^{-1}$$

The theoretical specific capacity (C_t) of the **Melem-PDI** polymeric network for Mg system, considering each repeating unit contribute to a 1-electron (1e$^-$ from half PDI) redox process, assuming no contribution from the hepazinne unit:

$$C_t = \frac{nF\ (C * mol^{-1})}{mol\ wt\ (g * mol^{-1})}$$

$$= \frac{1 * 96500\ C}{251.21\ g}$$

$$= \frac{1 * 96500\ mAh}{251.21\ g * 3.6}$$

$$= 106.71\ \text{mAhg}^{-1}$$

Cyclic voltammograms for Li battery

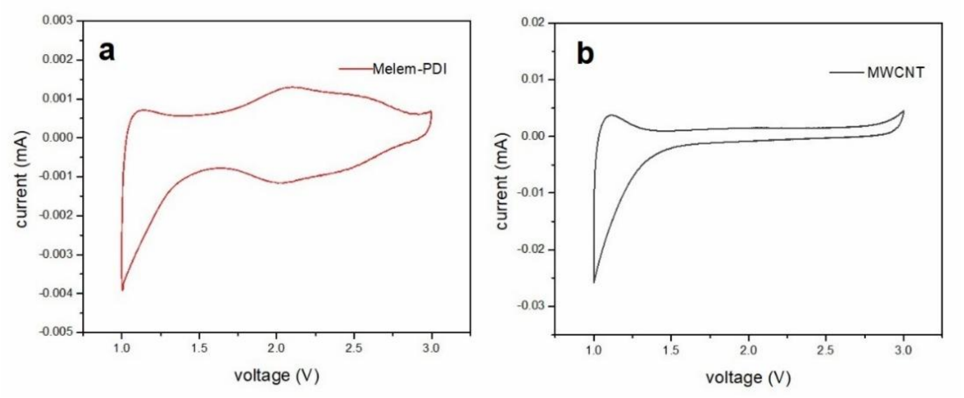

Figure S18: CV profiles of a) **Melem-PDI** and b) **MWCNT** against Li at a scan of 0.1 mV s^{-1}.

Electrochemical kinetic experiment for Li battery

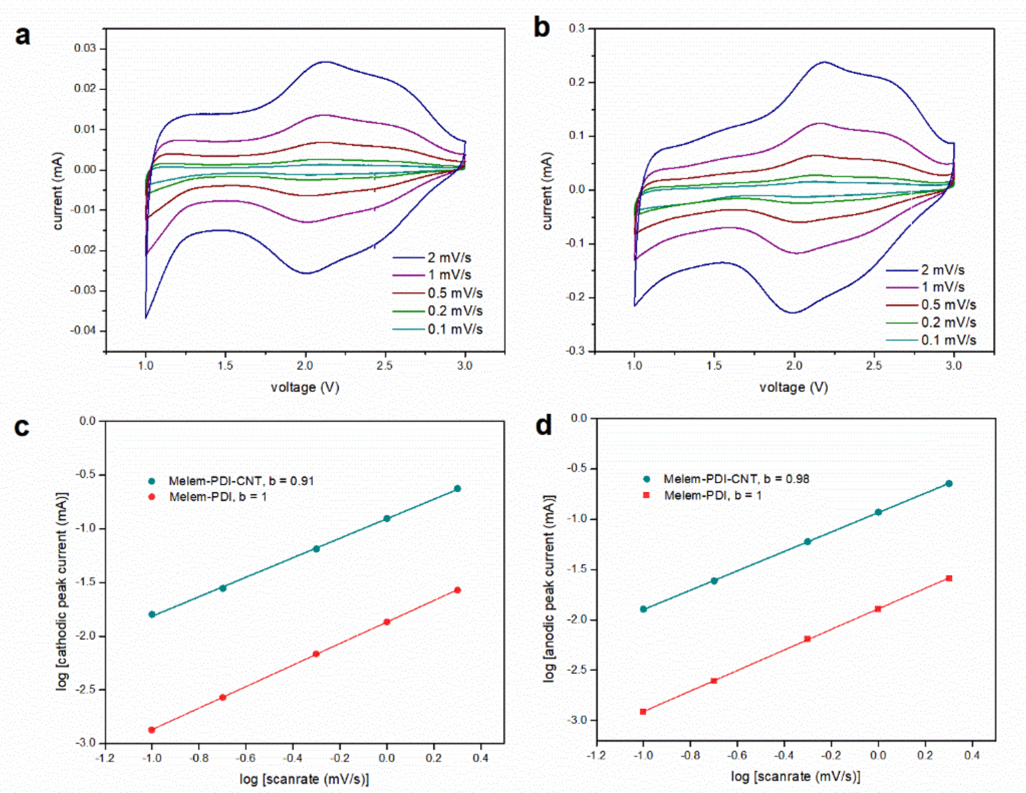

Figure S19: CV profiles of a) **Melem-PDI** and b) **Melem-PDI-CNT** at various scan rates against Li. log-log plot of c) cathodic peak current and d) anodic peak current, vs scan rate obtained from the CV.

Post cycling FT-IR analysis on electrode

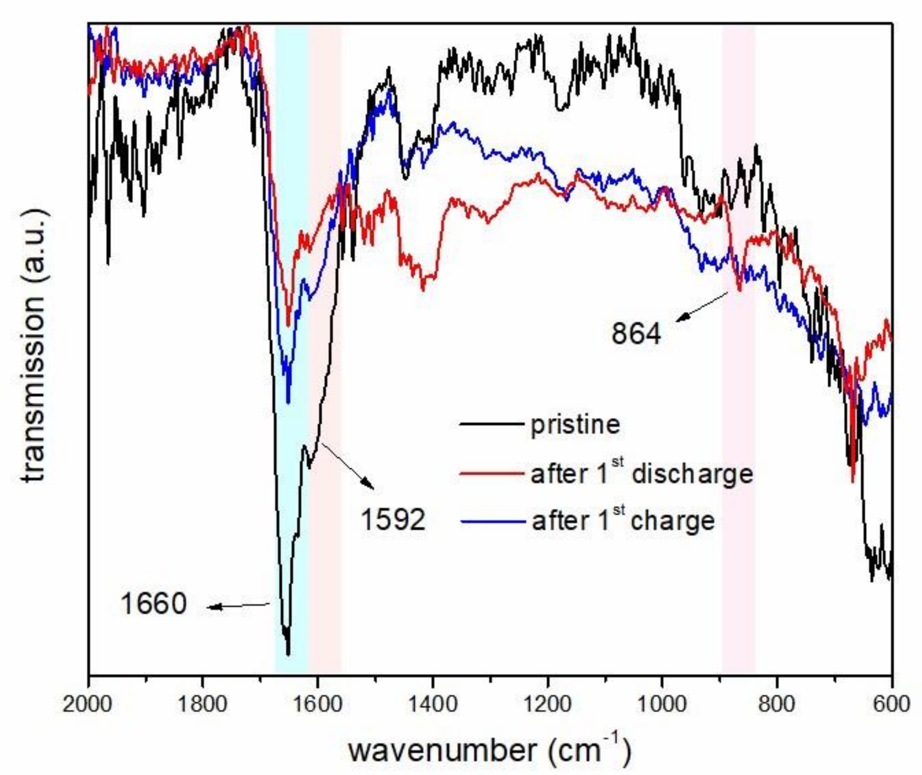

Figure S20: *Ex situ* FT-IR spectra of electrodes: pristine electrode (black), after 1st discharge (red) and after 1st charge (blue).

Cyclic voltammograms for Mg battery

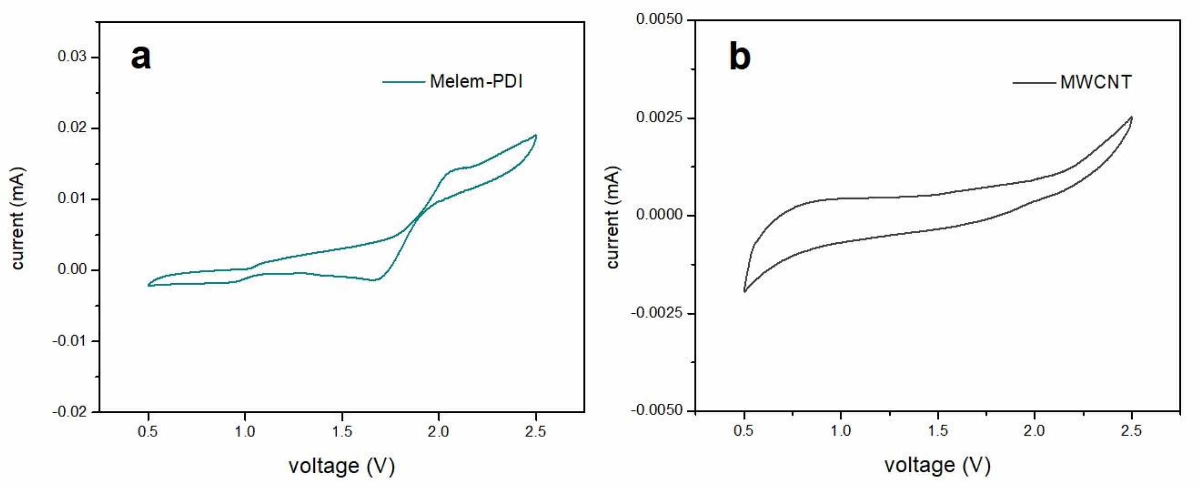

Figure S21: CV profiles of a) **Melem-PDI** and b) **MWCNT** against Mg at a scan of 0.1 mV s^{-1}.

Electrochemical kinetic experiment for Mg battery

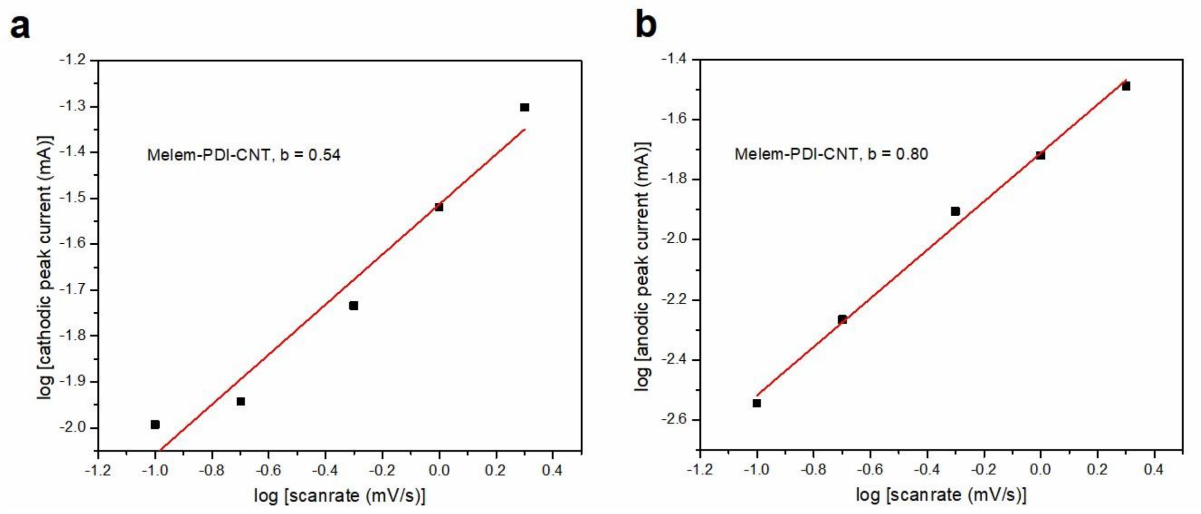

Figure S22: Log-log plot of c) cathodic peak current and d) anodic peak current, vs scan rate obtained from Figure 4a.

Battery cycling of Melem-PDI | Mg cell

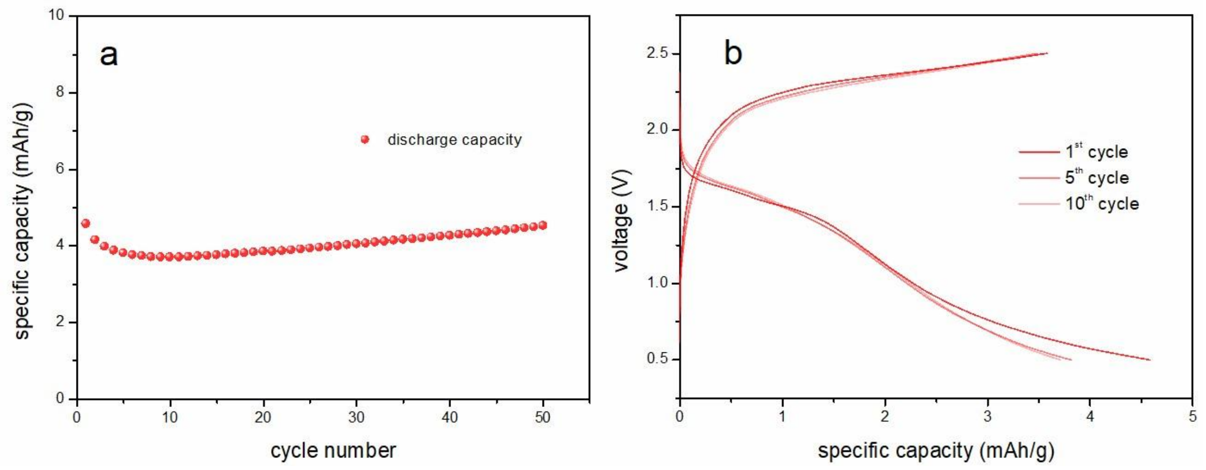

Figure S23: a) Battery cycling performance of **Melem-PDI** | Mg cell at a current rate of 200 mA g^{-1}, b) galvanostatic charge-discharge profile of **Melem-PDI** | Mg cell for selected cycles.

Summary of elemental and XPS analysis

Table S1. Elemental compositions (CHN) using elemental analysis.

Sample	Composition (wt.%)			
	C	H	N	C/N ratio
**Melem**	29.98	3.01	57.35	0.52
**Melem-PDI**	58.02	2.17	10.40	5.58
**Melem-PDI-CNT**	65.67	1.31	6.19	10.61

Table S2. Elemental compositions by using XPS analysis.

Sample	C, at.%	N, at.%	O, at.%	C/N ratio
**Melem**	43.8	51.9	3.7	0.84
**PTCDA**	80.6	-	19.0	-
**Melem-PDI**	67.0	14.1	12.2	4.75
**Melem-PDI-CNT**	73.6	9.6	12.0	7.67

Table S3. Surface nitrogen content and nitrogen functionalities (Pyridinic–N, Amide–N, Imide–N, Graphitic–N and Pyridinic –N^+-O_x^-) using XPS analysis.

Sample	Surface N content (at.%)	Pyridinic -N/N-C-NH_2 (fract.%)	Amide-N/N-C-N-C=O (fract.%)	Imide-N (fract.%)	Graphitic-N (fract.%)	Pyridinic -N^+-O_x^- (fract.%)
**Melem**	51.9	72.2	17.7	-	6.1	4.0
**Melem-PDI**	14.1	12.7	55.5	3.0	27.2	1.6
**Melem-PDI-CNT**	9.6	21.6	47.9	6.8	21.7	2.0

Table S4. Surface oxygen content and oxygen functionalities using XPS analysis.

Sample	Surface O content (at.%)	C-O-C (fract.%)	C=O (fract.%)
**PTCDA**	19.0	44.4	55.6
**Melem-PDI**	12.2	25.6	74.4
**Melem-PDI-CNT**	12.0	26.7	73.3

Literature comparison

Table S5: Performance comparison of current work with previously reported similar materials for Li-battery

Structure of PDI Frameworks and small molecules,	C_{theor} (mAhg^{-1})	Electrolyte	Capacity (mAhg^{-1}), current density	Potential: oxidation/ reduction (V), reference	Cycling stability: retention, cycles, current density	Ref
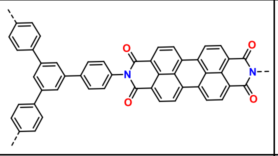	109.2	1 M, $LiPF_6$, EC:EMC: DMC 1:1:1 (v/v/v)	78.1, 25 mAg^{-1}	~2.35, Li/Li$^+$	74.1%, 65, 25 mAg^{-1}	1
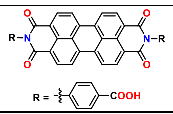	85	NR	85, 1 C; 68, 10 C	2.7-2.0, Li/Li$^+$	88%, 200, 5 C	2
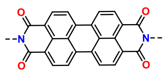	128.5	1 M $NaPF_6$, PC	126, 100 mAg^{-1}; 94.5, 800 mAg^{-1}	2.75-1.97, Na/Na$^+$	~90%, 50, 100 mAg^{-1}	3
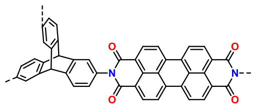	96.4	1 M, $LiPF_6$, EC:DMC 1:1 (v/v)	75.9, 0.05 C	~2.5 V, Li/Li$^+$	80.2%, 500, 2 C	4
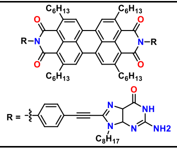	37	1 M, $LiPF_6$, EC:DMC 1:1 (v:v)	27, 0.05 C	~2.0 (2.3 & 1.8) V, Li/Li$^+$	99.97%, 300, 0.05 C	5
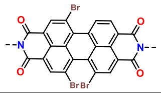	86	1 M $NaPF_6$, PC	78, 0.25 C	~2.0V, Na/Na$^+$	93%, 20, 0.125 C	6
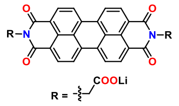	103	1 M, $LiPF_6$, EC:DMC 1:1 (v/v)	102, 17 mAg^{-1}	2.0 & 2.25V, Li/Li$^+$	86%, 1000, 0.2 mAg^{-1}	7
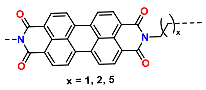	120	1 M, $LiPF_6$, EC:DMC 1:1 (v/v)	100, 5 Ag^{-1}	NR	86% 1000, 1 Ag^{-1}	8
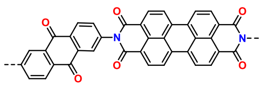	135	1 M $LiN(CF_3SO_2)$ DOL:DME 2:1 (v:v)	128.4, 50 mAg^{-1}	~1.1V, Li/Li$^+$	90%, 280, 200 mAg^{-1}	9
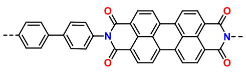	99	1M, $LiPF_6$, EC:DMC 1:1 (v:v)	120, 25 mAg^{-1}	~1.84 V, Li/Li$^+$	61%, 10, 200 mAg^{-1}	10
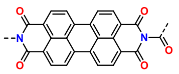	124	1M, $LiPF_6$, EC:DMC 1:1 (v:v)	119, 25 mAg^{-1}	~2.1 V, Li/Li$^+$	99.9%, 10, 200 mAg^{-1}	10

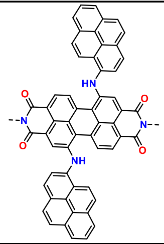	65.46	1 M, $LiPF_6$, EC:DMC 1:1 (v:v)	51, 25 mAg^{-1}	2.25-2.65V, Li/Li^+	35.3%, 50, 25 mAg^{-1}	11
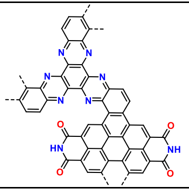	268.33	-	103, 5 C	-	10000, 20 Ag^{-1}	12
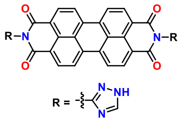	102	1 M $LiPF_6$, EC:DMC 1:1 (v:v)	85.2, 0.05 Ag^{-1}	1.5−3.5 V, Li/Li^+	84%, 100, 0.05 Ag^{-1}	13
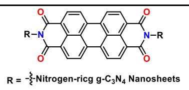 *Anode material*	524	-	587, 1 Ag^{-1}	-	99%, 250, 1 Ag^{-1}	14
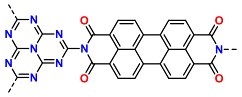 **Melem-PDI**	213	1M LiTFSI + 0.25 M $LiNO_3$ DOL/DME 1:1 (v:v)	77, 50 mAg^{-1} @500th cycle	~2 V, Li/Li^+	-	***This work***
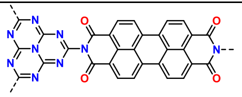 MWCNT (30 Wt %) **Melem-PDI-CNT**	213	1M LiTFSI + 0.25 M $LiNO_3$ DOL/DME 1:1 (v:v)	151, 50 mAg^{-1} @500th cycle	~2 V, Li/Li^+	85%, 5000, 0.05 Ag^{-1}	***This work***

Table S6: Performance comparison of current work with previously reported similar materials for Mg-battery

**Structure of Organic cathode Material use in Mg batteries**	**Electrolyte**	**Capacity ($mAhg^{-1}$), current density**	**Potential (vs Mg): oxidation/ reduction (V)**	**Cyclability feature ($mAhg^{-1}$), cycles, current density**	**Ref**
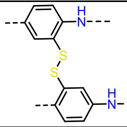	0.25 M $MgAlCl_3Bu_2$/THF	50, 25 mAg^{-1}	0.3 – 1.8	65, 33, 25 mAg^{-1}	15
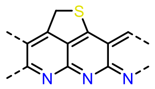	0.25 M $Mg(AlCl_2BuEt)_2$/THF	117.3, 25 mAg^{-1}	0.3 – 2.0	73, 22, 25 mAg^{-1}	15
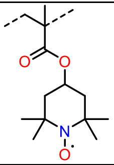	0.25 M $Mg(AlCl_2BuEt)_2$/THF	81.2, 22.8 mAg^{-1}	0.3 – 1.8	23, 21, 22.8 mAg^{-1}	16
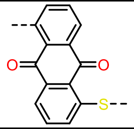	0.37 M $MgCl_2$+0.15 M $Mg(TFSI)_2$ in THF/glyme	225, 50 mAg^{-1}	0.5 – 2.5	60, 100, 50 mAg^{-1}	17

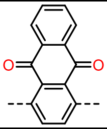	0.3 M $Mg(HMDS)_2$-$4MgCl_2$/THF	122, 130 mAg^{-1}	0.5 – 2.5	100, 100, 130 mAg^{-1}	18
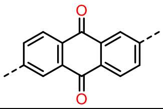	0.3 M $Mg(HMDS)_2$-$4MgCl_2$/THF	132.7, 130 mAg^{-1}	0.5 – 2.5	104.9, 100, 130 mAg^{-1}	18
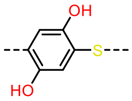	0.48 M $MgCl_2$+0.32 M $Mg(TFSI)_2$ /tetraglyme: DOL, 4:5	75, 50 mAg^{-1}	0.8 – 3.0	140, 25, 50 mAg^{-1}	19
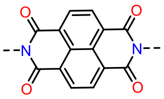	0.4 M $Mg(TFSI)_2$ / 0.4 M MgCl2 in tetraglyme: DME:DOL	45, 50 mAg^{-1}	0.8 – 2.5	85, 100, 50 mAg^{-1}	20
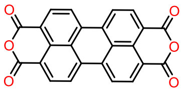	0.25 M APC+LiCl	126, 200 mAg^{-1}	0.6 – 2.5	100, 150, 200 mAg^{-1}	21
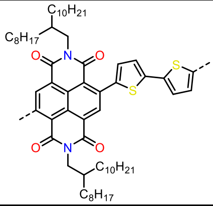	0.2 M $Mg(TFSI)_2$ in diglyme	49, 300 mAg^{-1}	0.8 – 2.0	43, 2500, 300 mAg^{-1}	22
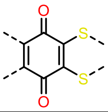	0.4 M $Mg(TFSI)_2$ in sulfolane	52, 0.05 C	1.3 – 2.9	63, 45, 0.05 C	23
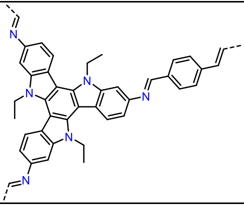	0.3 M $Mg[B(hfip)_4]_2$·3DME in DME	40, 5 C	2.0 – 3.8	33.6, 1000, 5 C	24
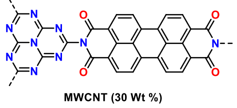 **Melem-PDI-CNT**	0.4 M $Mg[B(hfip)_4]_2$ in DME	40, 200 mAg^{-1}	0.5 – 2.5	23, 500, 200 mAg^{-1}	***This work***

Abbreviation: EC- ethylene carbonate; DMC- dimethyl carbonate; DEC- diethyl carbonate; PC- propylene carbonate; DOL- dioxolane; DME- 1,2-dimethoxyethane; LiPF6- lithiumhexafluorophosphate; NaPF6- sodium hexafluorophosphate; LiTFSI- lithium bis(trifluoromethanesulfonyl)imide; APC- phenyl complex; $Mg(HMDS)_2$- Magnesium-bis(hexamethyldisilazid); $Mg(TFSI)_2$- Magnesium bis(trifluoromethanesulfonimide); $Mg[B(hfip)_4]_2$-(magnesium tetrakis(hexafluoroisopropyloxy) borate).

References

1) D. Tian, H. Z. Zhang, D. S. Zhang, Z. Chang, J. Han, X. P. Gao, X. H. Bua, *RSC Adv.* **2014**, *4*, 7506.

2) M. E. Bhosale, K. Krishnamoorthy, *Chem. Mater.* **2015**, *27*, 2121.

3) H. Banda, D. Damien, K. Nagarajan, M. Hariharan, M. M. Shaijumon, *J. Mater. Chem. A* **2015**, *3*, 10453.

4) T. B. Schon, A. J. Tilley, E. L. Kynaston, D. S. Seferos, *ACS Appl. Mater. Interfaces* **2017**, 9, 18, 15631.

5) Y. L. Wu, N. E. Horwitz, K. S. Chen, D. A. G. Gualdron, N. S. Luu, L. Ma, T. C. Wang, M. C. Hersam, J. T. Hupp, O. K. Farha, R. Q. Snurr, M. R. Wasielewski, *Nat. Chem.,* **2016**, 9, 466.

6) H. Banda, D. Damien, K. Nagarajan, A. Raj, M. Hariharan, M. M. Shaijumon, *Adv. Energy Mater.,* **2017**, 1701316.

7) M. Veerababu, R. Kothandaraman, *Electrochimica Acta* **2017**, 232, 244–253.

8) D. Wu, G. Zhang, D. Lu, L. Ma, Z. Xu, X. Xi, R. Liu, P. Liu, Y. Su, *J. Mater. Chem. A* **2018***, 6, 13613.*

9) M. H. Jung, R. V. Ghorpade, *J. Electrochem. Soc.,* **2018**, 165, 2476.

10) M. R. Raj, R. V. Mangalaraja, D. Contreras, K. Varaprasad, M. V. Reddy, S. Adams*, ACS Appl. Energy Mater.* **2020**, *3*, 1, 240.

11) M. R. Raj, R. V. Mangalaraja, G. Lee, D. Contreras, K. Zaghib, M. V. Reddy, *ACS Appl. Energy Mater.* **2020**, *3*, 7, 6511–6524.

12) J. C. Russell, V. A. Posey, J. Gray, R. May, D. A. Reed, H. Zhang, L. E. Marbella, M. L. Steigerwald, Y. Yang, X. Roy, C. Nuckolls, S. R. Peurifoy, *Nat. Mater.* **2021***, 20, 1136.*

13) H. Seong, W. Nam, J. H. Moon, G. Kim, Y. Jin, H. Yoo, T. Jung, Y. Myung, K. Lee, J. Choi, *ACS Appl. Mater. Interfaces.* **2023***, 15, 58451.*

14) M. R. Raj, J. Yun, D. Son, G. Lee, *Energy Environ. Mater.* **2023**, *6*, e12553.

15) Y. NuLi, Z. Guo, H. Liu, J. Yang, *Electrochem. Commun.* **2007**, *9*, 1913.

16) C. Qiang, N. Y. Na, G. Wei, Y. Jun, W. J. Lin, G. Y. Guo, *Acta Phys. Chim. Sin.* **2013**, *29*, 2295.

17) J. Bitenc, K. Pirnat, T. Bančič, M. Gaberšček, B. Genorio, A. R. Vitanova, R. Dominko, *ChemSusChem* **2015**, *8*, 4128.

18) Z. Song, Y. Qian, M. L. Gordin, D. Tang, T. Xu, M. Otani, H. Zhan, H. Zhou, D. Wang, *Angew. Chem. Int. Ed.* **2015**, *54*, 13947.

19) J. Bitenc, K. Pirnat, G. Mali, B. Novosel, A. R. Vitanova, R. Dominko, *Electrochem. Commun.* **2016**, *69*, 1.

20) T. Bančič, J. Bitenc, K. Pirnat, A. Kopač Lautar, J. Grdadolnik, A. R. Vitanova, R. Dominko, *J. Power Sources* **2018**, *395*, 25.

21) L. Cui, L. Zhou, K. Zhang, F. Xiong, S. Tan, M. Li, Q. An, Y. M. Kang, L. Mai, *Nano Energy* **2019**, *65*, 103902.

22) H. Dong, Y. Liang, O. Tutusaus, R. Mohtadi, Y. Zhang, F. Hao, Y. Yao, *Joule* **2019**, *3*, 782.

23) N. A. Tran, J. C. Lepretre, F. Alloin, *Electrochim. Acta* **2021**, *375*, 137990.

24) S. M. Pallasch, M. Bhosale, G. J. Smales, C. Schmidt, S. Riedel, Z. Z. Karger, B. Esser, O. Dumele, *J. Am. Chem. Soc.* **2024**, *146*, 17318.
